# Identification and Validation of a Prognostic Signature Based on Fibroblast Immune-related Genes to Predict the Prognosis and Therapeutic Response of renal clear cell carcinoma by Integrated Analysis of Single-Cell and Bulk RNA Sequencing Data

**DOI:** 10.7150/jca.100194

**Published:** 2024-09-23

**Authors:** Shuwen Zhang, Yuqian Yang, Liyi Zhang, Yijiang Liu, Zihun Guo, Jiajun Wu, Weijun Zhou, Zhengdong Hong, Wenxiong Zhang

**Affiliations:** 1 Department of Thoracic Surgery, The Second Affiliated Hospital, Jiangxi Medical College, Nanchang University, Nanchang, China, 330006.; 2 Queen Mary School, Jiangxi medical college, Nanchang University, Nanchang, China, 330006.; 3 Department of Urology Surgery, The Second Affiliated Hospital, Jiangxi Medical College, Nanchang University, Nanchang, China, 330006.; 4 Jiangxi medical college, Nanchang University, Nanchang, China, 330006.

**Keywords:** Fibroblast, Immune-related genes, Kidney renal clear cell carcinoma, Prognosis model, Single cell analysis.

## Abstract

**Background**: The importance of fibroblasts in cancer progression is becoming more acknowledged, particularly the significance of their immune-related genes. However, the precise roles these genes play in fibroblasts throughout tumor development remains unclear. Exploring how these genes function in advancing kidney renal clear cell carcinoma (KIRC) could provide answers to these uncertainties.

**Material and method**: The Cancer Genome Atlas (TCGA) database served as the source of data for KIRC patients. We distinguished fibroblast immune-related genes (FIGs), which are used to construct risk score. Further analysis conducted including enrichment analysis, assessment of tumor mutation burden (TMB), evaluation of tumor microenvironment (TME), analysis of immune cell infiltration, and drug sensitivity prediction.

**Result**: The risk score using 6 FIGs effectively predicts the outcomes for KIRC patients. Nomogram which is based on the risk score and clinical data, demonstrated superior predictive performance compared to the version without the risk score. Enrichment analysis identified that coagulation pathway predominates in high-risk group, the protein secretion pathway is prevalent in low-risk patients' cohort. The adverse prognosis in high-risk patient cohort could be linked to an elevated TMB. TME analysis showed that high-risk group's tumor tissues contain more immune and stromal cells. Furthermore, the amount of regulatory T cells increases with the risk score. Low-risk group response better to immunotherapy. Finally, RT-qPCR confirmed the differential expression of FIGs in KIRC patients.

**Conclusion**: This risk score and nomogram are valuable tools assessing KIRC patients' prognosis. Poorer prognosis in high-risk categories may have relationship with activation of coagulation pathway and a higher TMB.

## Introduction

Renal cell cancer (RCC) is among the most prevalent genitourinary tract malignancies, exhibiting a mortality rate of 30% to 40% 1. KIRC is its most common subtype, though its prognosis often proves challenging to determine 2. While the American Joint Committee on Cancer put forward a tumor, node, metastasis (TNM) classification system for KIRC prognosis assessment, categorizing patients into stages I through IV 3. Currently, prognostic markers for KIRC typically depend on imaging techniques, which lack high specificity 4. This underscores the critical need for developing new, more effective prognostic models for patients with KIRC.

Fibroblasts, the predominant cells in connective tissue, are traditionally recognized for their significant contributions to wound healing and tissue repair 5. However, nowadays' research has revealed that fibroblasts also play vital apart in the progression of several cancers, such as lung, breast, and kidney cancers 6-8. Within TME, fibroblasts have been recognized to provoke tumor cell growth, proliferation, and metastasis 9. Muhammad Khan *et al.* (2022) and Song-Chao Li *et al.* (2022) established distinct KIRC prognosis models employing pyroptosis relates genes and telomere-related genes respectively 10, 11. Furthermore, Yixin Liu *et al.* (2023) created a model that uses cuproptosis-related genes to predict the prognosis for KIRC patients 12. Single-cell analysis serves as a crucial tool for examining cellular heterogeneity within complex biological systems. This technique enables the identification of the full gene expression profile of individual cells, thereby aiding in the discovery of genes previously unidentifiable 13. In recent studies, Jiayu Zhang *et al.* (2023) sought to propose a pan-cancer prognostic model by analyzing the single cell data of endothelial cells 14. Similarly, Jing Zhang *et al.* (2023) explored the use of ferroptosis-associated genes for determining the prognosis of KIRC patients using single-cell data 15. However, the prognostic significance of FIGs in KIRC still remains to be fully elucidated.

Consequently, we developed a risk score for KIRC centered on FIGs, designed to evaluate KIRC patients' prognosis. We further examined clinical applicability of this risk score by develop a nomogram. Through enrichment analysis, differential analysis of the TME, and TMB analysis, we want to find out the pathway involved. Drug sensitivity prediction can give clues for clinical personalized medicine.

## Material and method

### Data sources

We obtained transcriptome data from TCGA database (https://portal.gdc.cancer.gov/, as of January 25, 2024) for 614 samples, representing 533 KIRC patients, which included 533 tumor and 77 normal tissue samples. Additionally, we downloaded clinical pathologic data for 537 KIRC patients from the same database. We collected 2484 genes linked to immunity from the ImmPort online database (https://www.immport.org/, as of February 26, 2024) and 3714 genes which is related to immune response from the InnateDB website database (https://www.innatedb.ca/, as of February 26, 2024). After eliminating duplicates, we compiled 2533 unique immune related genes. Genomic mutation data for the tumor cells were also sourced from the TCGA. Furthermore, Tumor Immune Single-cell Hub 2 (TISCH2) database provided us with single cell data (http://tisch.comp-genomics.org/, as of February 26, 2024; sample ID: T010042; dataset name: KIRC-GSE111360).

### Finding the genes that are differently expressed in fibroblasts

We acquired data of every single cell differentially expressed genes from the TISCH2 database. To isolate those genes which were significantly differentially expressed between other cell types and fibroblasts, we specifically analyzed the fibroblasts cell gene expression profile. Genes with a fold change more than 1.5 and an adjusted p-value less than 0.05 were our selections 14.

### Construction of risk score

To pinpoint FIGs, we conducted an intersection analysis between known immune-related genes and the differentially expressed genes in fibroblasts, thereby identifying FIGs. We utilized the “VennDiagram” R package to graphically represent this intersection through a Venn diagram. Further, to explore the interactions among these identified genes, we uploaded the genes data set to STRING website database (https://cn.string-db.org/, as of February 26, 2024), where we set the minimum interaction score to 0.4. We were able to create a protein-protein interaction (PPI) network as a result. From this network relationship file downloaded from STRING, we identified genes that interact with more than 20 neighboring nodes, considering these as “most core” genes within the network. The gene with the highest number of adjacent interacting neighbors was designated as the core gene, potentially playing a momentous role in the contribution of fibroblasts for KIRC.

Initially, to create the risk score, every KIRC patient was divided into testing and training groups at random. We then operated univariate Cox regression analysis (p<0.05) by using the “survival” and “survminer” R packages to find genes linked to patient prognosis. Significant genes from this analysis were further refined through Least Absolute Shrinkage and Selection Operator (LASSO) regression by the usage of “glmnet” R package, aiming to eliminate the risk of overfitting. Finally, genes that were most closely associated with prognosis were selected via multivariate Cox regression for constructing risk score 12. Risk score was established as follows: Risk score = ∑ (coefficient of gene n) × (expression of gene n). Based on the median of the risk score, patients, including those in the train and test group, were then separated into high-risk and low-risk categories.

### Risk score accuracy validation

To validate the accuracy of our risk score, the “survival” and “survminer” R packages were utilized to perform Kaplan-Meier (K-M) survival analysis. This enabled the examination of differences in overall survival (OS) between different groups. Progression-free survival (PFS) curves were also generated. Furthermore, we made use of the R package “pheatmap” to create heatmaps of the risk scores for all patients, and those in the training and testing groups. These heatmaps made it easier to explore the connection between patient survival status and risk genes expression, enhancing our understanding of the model's construction principles. To delve deeper into the influence of specific genes on patient survival, we plotted survival curves for each gene and analyzed the association between these risk genes' expression and the risk scores.

Principal Component Analysis (PCA) was conducted using all FIGs and risk genes respectively to appraise the effectiveness of the grouping. Additionally, both multivariate and univariate Cox independent prognostic analysis help deciding the prognostic contribution of the risk score, with results presented in a forest plot. We also utilized the “timeROC” R package to draw 1, 3, and 5-year's Receiver Operating Characteristic (ROC) curves, calculating the area under the curve (AUC) to measure the predictive efficacy of risk score. Furthermore, validation of risk score within clinical subgroups, and clinical relevance analysis were carried out to deepen our understanding of the connection between risk score and various clinical phenotype.

### Nomogram plotting to predict KIRC patients' prognosis

By using the “RMS” R package, we developed a nomogram that predict the 1, 3, and 5-year survival rates based on five key factors: gender, tumor grade, age, risk score, and tumor stage. This tool aims to provide precise predictions of patient outcomes. To assess the accuracy and utility of the nomogram, we generated calibration curves for the 1, 3, and 5-year. In addition, we employed the “ggDCA” package to produce decision curves, evaluating the clinical usefulness of the nomogram in making clinical decisions. The predictive accuracy of the nomogram was further analyzed by drawing ROC curves and calculating their AUC. To underscore the prediction efficacy of our nomogram has exceed traditional clinical data, we also plotted C-index curves.

### Enrichment analysis to find relevant pathways

We acquired five representative gene sets which come from the Gene Set Enrichment Analysis (GSEA) database (https://www.gsea-msigdb.org/, as of February 26, 2024), and carried out enrichment analysis. To dig deeper into the biological pathways and functions related to the differentially expressed genes, we use the “ClusterProfiler” R package, and carried out enrichment analysis for Gene Ontology (GO) and the Kyoto Encyclopedia of Genes and Genomes (KEGG). The results of GO enrichment analysis were annotated utilizing Bioconductor annotation package “org.Hs.eg.db” 10.

### Tumor microenvironment and immune-related analysis

We employed the “estimate” R package for analyzing the TME of samples from high-risk and low-risk groups, creating violin plots to compare the infiltration of non-tumor cells between these groups. For a more detailed examination of immune cell composition variations, we used the “CIBERSORT” R package to distinguish the variances in 22 immune cell lineages between 2 risk categories. To assist with immune phenotyping, we used the “RColorBrewer” R package. Additionally, single-sample gene set enrichment analysis (ssGSEA) was executed via “GSVA” R package. It was applied to evaluate variations in immune functions across the different risk groups. These analyses aim to highlight disparities in immune functionality between 2 risk categories, laying the groundwork for potential immunotherapy approaches. Lastly, we accessed the TIDE database website (http://tide.dfci.harvard.edu/, as of February 26, 2024) to retrieve TIDE score files, comparing these scores across different risk groups. This comparison aids in assessing the likely responsiveness of patients in different risk groups to immunotherapy.

### Tumor mutation burden calculation

We processed tumor mutation data from TCGA database utilizing the “TCGAbiolinks” R package and used “maftools” R package for generating waterfall plots. These plots illustrate the variances in tumor mutation genes and their mutation rates between different risk groups. Additionally, we created violin plots to display the variations in TMB between 2 different risk groups. To further explore the impact of mutations on survival, we operated survival analysis for groups categorized by mutation level and risk.

### Drug sensitivity prediction

We utilized the “parallel” and “oncoPredict” R packages to predict and analyze drug sensitivity in patients within the different risk groups. Utilizing the “ggplot2” R package, we then created comparative box plots to display the sensitivity of these two groups to 198 different drugs. This analysis helped us identify drugs which have significant variances in sensitivity between 2 different risk groups, using a relative stringent significance threshold (p-value < 0.001).

### RT‒qPCR validation

The Typical Culture Preservation Commission Cell Bank of the Chinese Academy of Medical Sciences in Shanghai, China, provided the normal kidney epithelial cell lines (HK-2) and the KIRC cell lines (Caki-1 and Caki-2). HK-2 cells were cultured in Keratinocyte SFM (K-SFM) medium, whereas we grew Caki-1 and Caki-2 cells in a complete culture medium composed of 90% McCoy's 5a medium adding 10% fetal bovine serum. Trizol was employed to get total RNA extraction (Takara Bio, Inc., Otsu, Japan). Accurate Biology (Hunan, China) provided a reverse transcription kit for cDNA synthesis, which was followed by RT-qPCR using the SYBR Green premixed qPCR kit (Accurate Biology, Hunan, China) using a Roche LightCycler 480 II (Roche, Basel, China). We determined gene expression level through 2^-ΔΔCt method, we have shown the primer sequences in **Table S1**.

The Human Protein Atlas database (https://www.proteinatlas.org/) was utilized to assess the FIGs' expression at the protein level in both normal kidney tissues and KIRC tissues.

### Statistical analysis for result data

Result analysis for statistics was undertook by R software (version 4.3.0), combining with Perl (Strawberry Perl 5.30.0.1). To compare variables across groups, we used the Wilcoxon t-test; statistically significant p-value was one that was less than 0.05.

## Results

### Single-cell data analysis identifies genes with differential expression in fibroblasts

**Figure 1:**
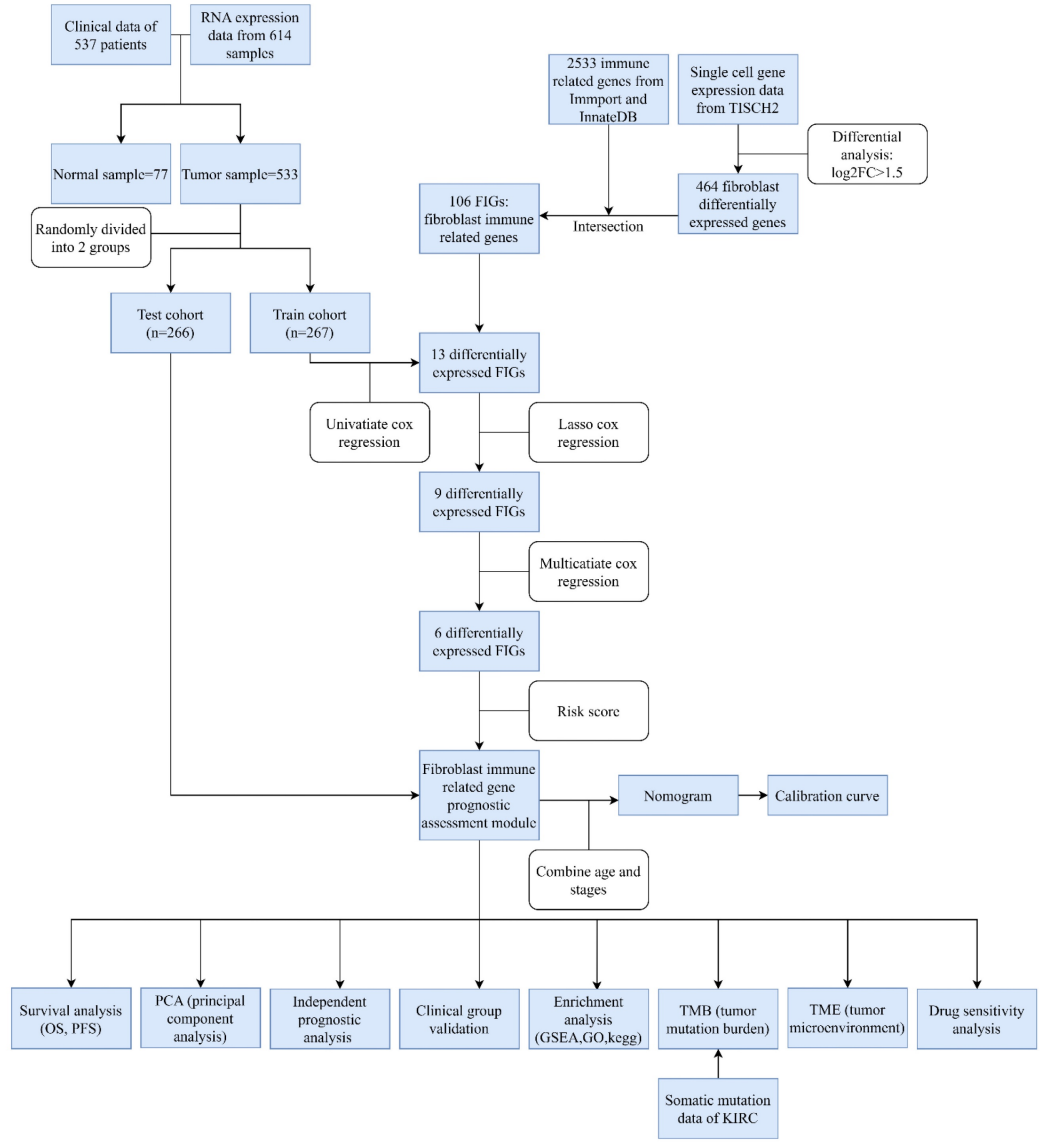
Flowchart.

The design of this study is depicted in a flowchart as shown in **Figure 1**. Single cell gene expression information for KIRC patients were retrieved from the TISCH2 database. Then using the filter condition mentioned in method part, we pinpointed 464 genes that exhibited significant differential expression in fibroblasts **(Table S2)**.

### Construction of the risk score

**Figure 2:**
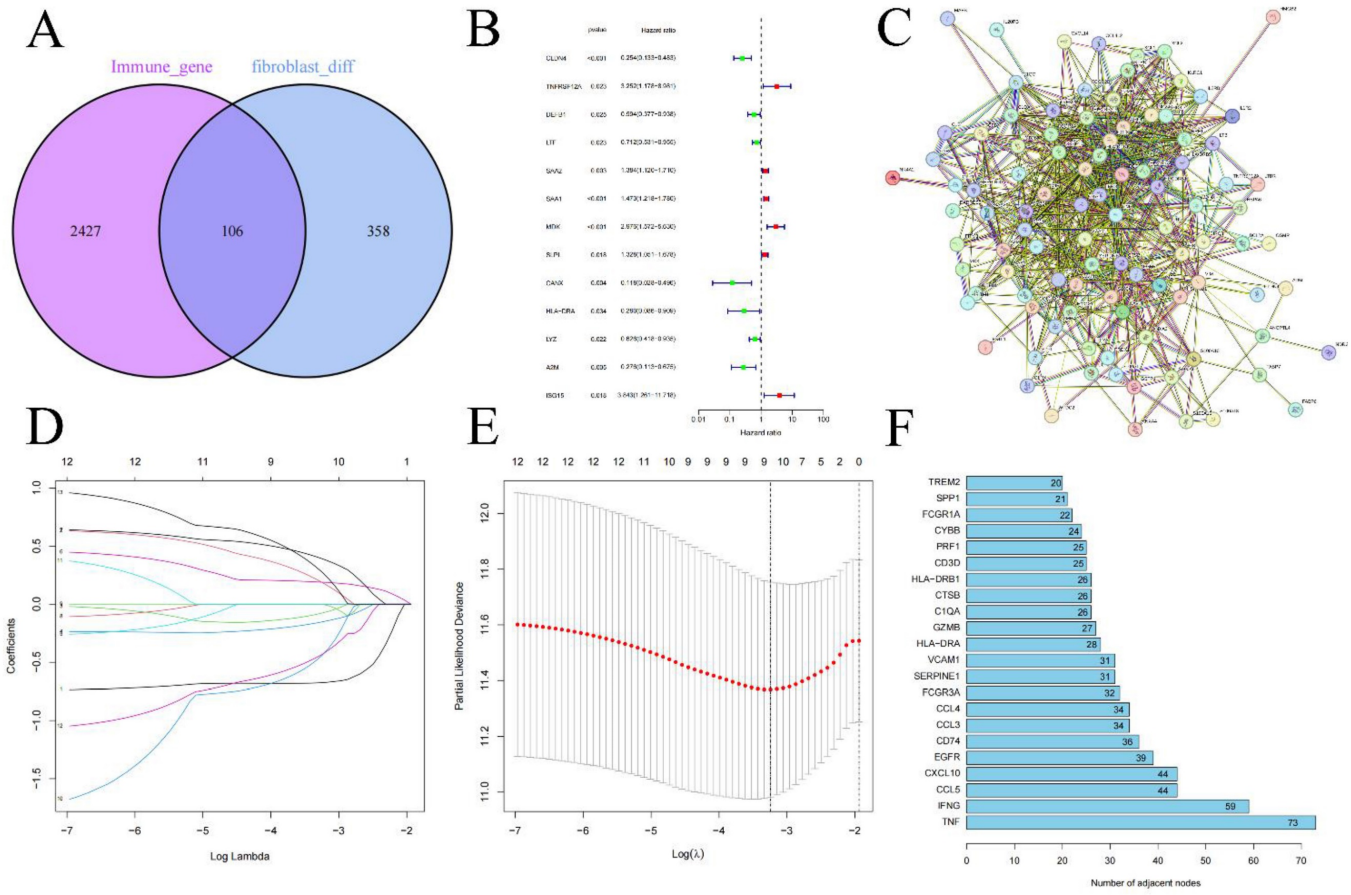
Intersection of immune-related genes and differentially expressed genes in fibroblasts resulted in 108 fibroblast immune-related genes **(A)**, differential genes obtained through univariate Cox regression analysis **(B)**, the protein interaction network of FIGs **(C)** and the screening of its core genes **(F)**, Lasso regression analysis **(D-E)**.

We performed an intersection analysis between the 464 differentially expressed fibroblast genes and 2533 genes which is related to immune response, 106 FIGs were identified as a consequence **(Figure 2A)**. These 106 genes were then uploaded to the STRING database to examine their interactions, which facilitated the construction of a PPI network **(Figure 2C)**. Further, we counted the number of adjacent nodes for each gene and found 22 genes with more than 20 neighboring nodes, for which we drew bar graphs of the number of adjacent nodes **(Figure 2F)**. We suspect that the tumor necrosis factor (TNF) gene, with 73 neighboring nodes, was the core gene among the FIGs.

Randomly, we split the 533 KIRC patients into two groups: a training group for risk score foundation and a testing group to verify it **(Table 1)**. Initially, through the use of univariate Cox regression analysis, 13 FIGs linked to prognosis were found, applying p-value less than 0.05 **(Figure 2B) (Table S3)**. Subsequently, we performed LASSO regression analysis, selecting 9 prognosis associated FIGs from this process **(Figures 2D, E)**. In the final step, multivariate Cox regression analysis helped us pinpoint 6 FIGs that were highly predictive of prognosis** (Table S4)**. We then used these six genes to create our KIRC risk score. The risk score calculation formula for every sample is: (CLDN4's expression × -0.667) + (LTF's expression × -0.318) + (SAA1's expression × 0.261) + (MDK's expression × 0.741) + (HLA-DRA's expression × -1.302) + (ISG15's expression × 0.793).

### Risk score accuracy verification

**Figure 3:**
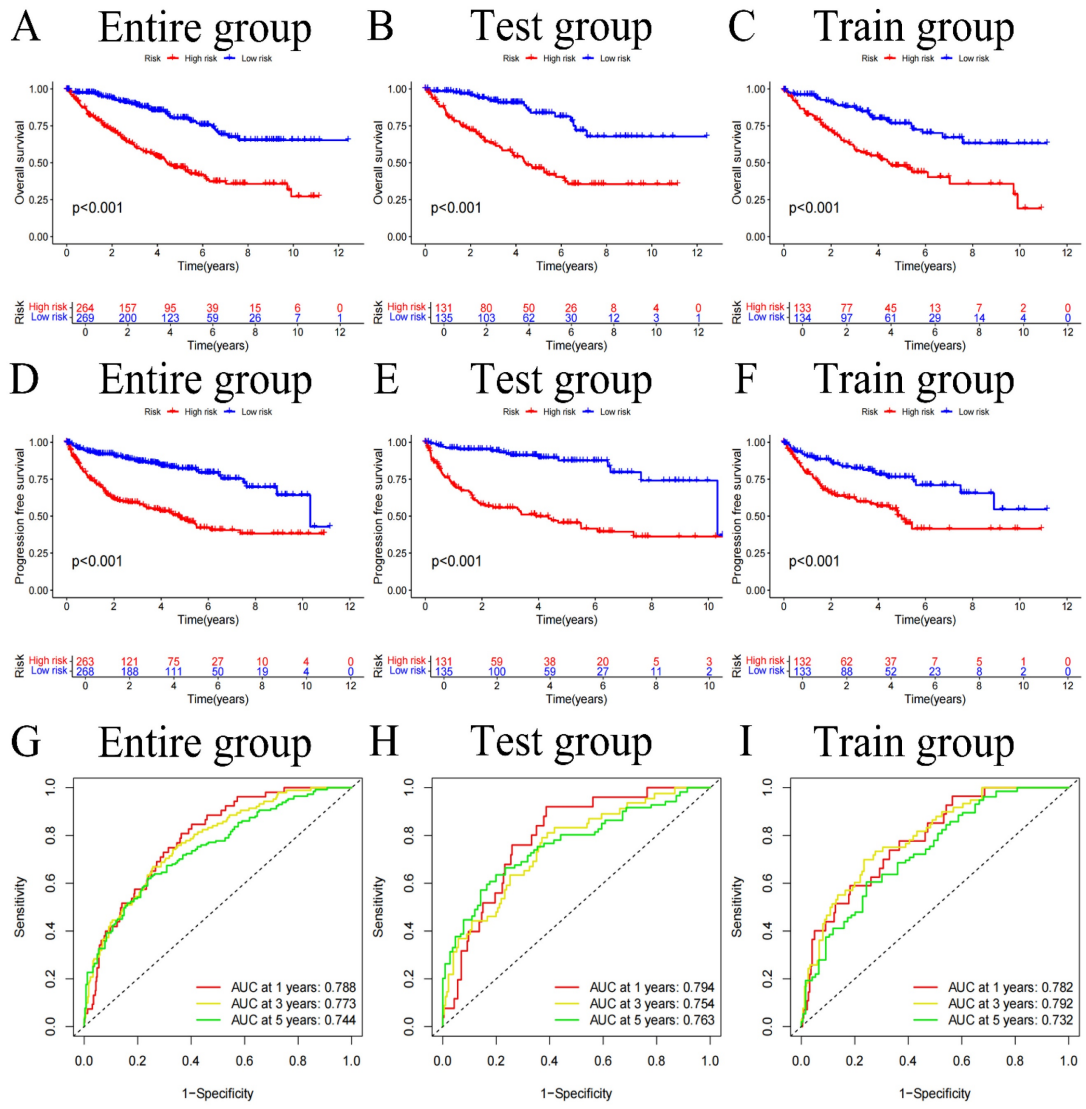
Overall survival (OS) curves for the entire group, test group, and train group **(A-C)**, Progression-free survival (PFS) curves **(D-F)**, and ROC curves **(G-I)**.

We divided the sample into high and low-risk groups based on the median value (0.938) of the risk score. After classifying the patient cohort into high-risk and low-risk categories** (Table 2)**, we conducted analysis on OS and PFS. Based on the findings, the low-risk cohort's survival status was much better than those of the high-risk (all p-values<0.001) **(Figure 3A-F)**. Additionally, a risk heatmap indicated that the low-risk cohort had higher levels of CLDN4, LTF, and HLA-DRA expression, identifying them as low-risk genes. The high-risk category exhibited elevated expression levels of SAA1, MDK, and ISG15, classifying them as high-risk genes **(Figure S1A-C)**. This observation was further substantiated by the relationship curves for 6 FIGs and risk scores** (Figure S2)**. According to scatter plots, patients in the high-risk category often had short survival times than low-risk patients **(Figure S1D-I)**. Additionally, to predict the influence of each FIGs on the prognosis of KIRC patients, survival curves were plotted for each FIGs** (Figure S3)**, showing that survival rates differed significantly even when considering the expression level of one FIGs (p-value<0.001). Subsequently to Cox independent prognostic multivariate and univariate analysis **(Figure S4A, B)**, the results highlighted several key independent risk factors. In the univariate analysis, age (with p-value<0.001, HR=1.031, CI=1.018-1.045), tumor grade (with p-value<0.001, HR=2.275, CI=1.859-2.785), tumor stage (with p-value<0.001, HR=1.859, CI=1.631-2.119), and risk score (with p-value<0.001, HR=1.408, CI=1.310-1.514) were all identified as independent risk factors. The multivariate analysis further confirmed aforemen-tioned 4 factors continued to be significant independent predictors of prognosis: age (with p-value<0.001, HR=1.033, CI=1.018-1.048), tumor grade (with p-value=0.009, HR=1.355, CI=1.077-1.705), tumor stage (with p-value<0.001, HR=1.601, CI=1.375-1.865), and risk score (with p-value<0.001, HR=1.285, CI=1.177-1.404). PCA was applied to confirm that our risk score effectively differentiates patients into distinct risk categories **(Figure S5H, I)**. Using this risk score, we forecast patient survival rates at 1, 3, and 5-year and generated the corresponding ROC curves** (Figures 3G-I)**. The AUC values for 1, 3, and 5-year are 0.788, 0.773, and 0.744 respectively, demonstrate the risk score's high efficacy in prognostic prediction. Further clinical relevance analysis revealed that our risk score accurately predicts various clinical features of patients. Significant variations in tumor grade, stage, and T and M stages were found between the 2 risk categories **(Figure S4C, D)** (all p-values<0.001), with box plots corroborating these findings **(Figure S5A-J)**. Survival curves segmented by clinical subgroups further underscored the validity of our risk score, demonstrating significantly lower survival times in high-risk cohort in contrast to low-risk** (Figure S6, Figure S7)** (all p-values<0.01).

### Nomogram construction

**Figure 4:**
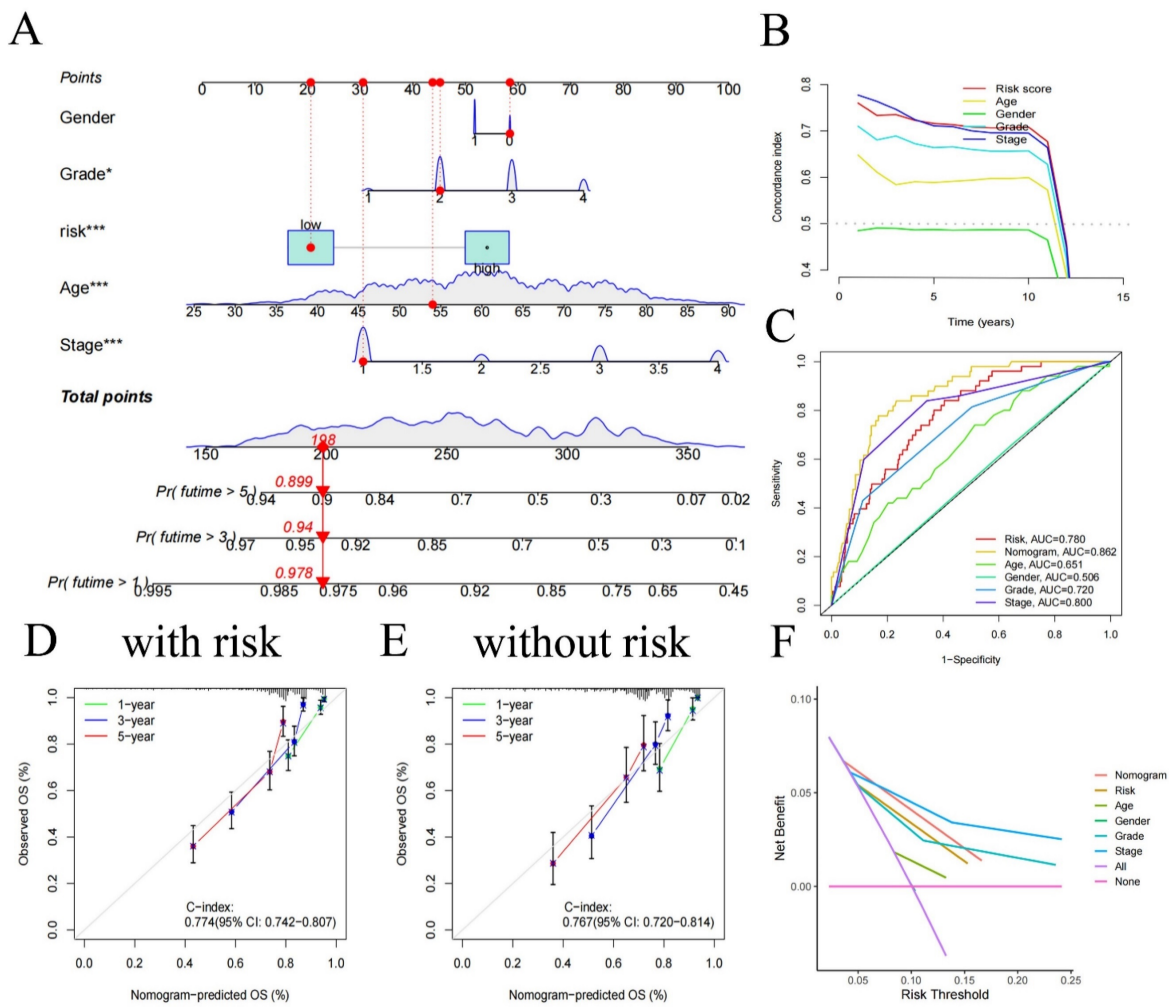
Nomogram constructed using age, gender, risk, and stage **(A)** and its C-index curve **(B)**, ROC curve **(C)**, calibration curve for the risk model **(D)**, and calibration curve for the no risk model **(E)**, decision curve **(F)**.

We created a nomogram **(Figure 4A)**, offering a device for predicting patients' clinical prognosis using our risk score together with patient clinical data (age, gender, grade, and stage). The calibration curves provided proof of the precision of the nomogram** (Figure 4D, E)**. To demonstrate the value of our nomogram in predicting patients' prognosis, we also plotted C-index curves, revealing the risk score's C-index (0.774) surpassed other clinical features', confirming our risk score has advantages in predicting patients' prognosis compared to other clinical traits **(Figure 4B)**. At the same time, we made 1, 3, 5-year ROC for all clinical traits **(Figure 4C)**, we found that the AUC of the nomogram (0.862 for 1-year, 0.825 for 3-year, 0.781 for 5-year) was higher than all clinical traits, indicating that using our nomogram to predict patient's prognosis is more accurate than any other single clinical trait. Decision curve analysis showed similar results** (Figure 4F)**, indicating significant practical value of our nomogram in clinical decision-making of patient's prognosis.

### Enrichment analysis to identify pathways involved

**Figure 5:**
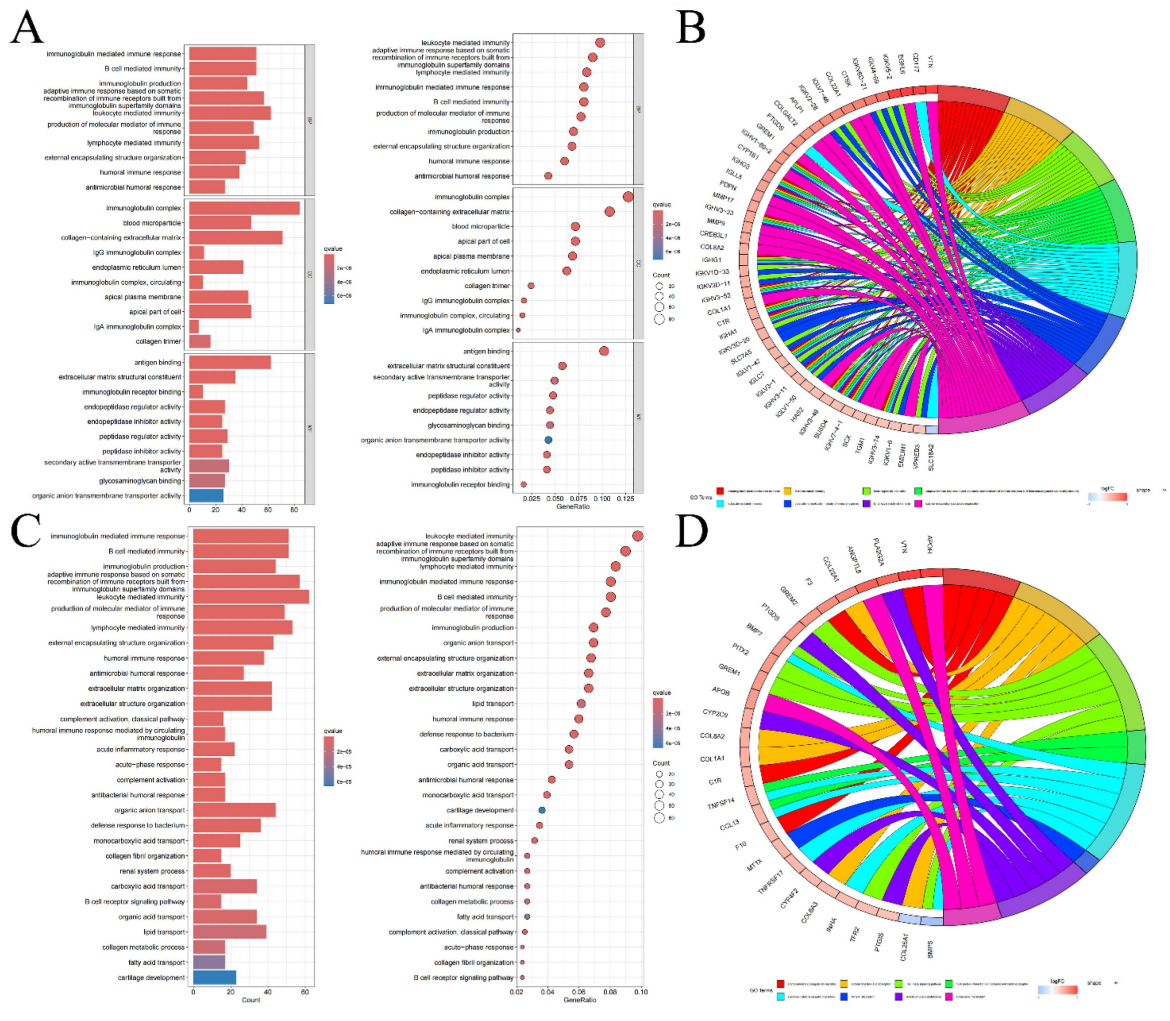
Bar graphs of GO enrichment analysis, bubble chart **(A)** and circle chart **(B)**. Bar graphs of KEGG enrichment analysis, bubble chart **(C)**, and circle chart **(D)**.

We conducted GSEA using five representative gene list files and identified the top 5 pathways concentrated in both high-risk and low-risk groups, as outlined in **Table S5** and visualized in **Figure S8**. According to the GSEA results, in the high-risk category, genes differentially expressed were mostly concentrated in routes connected to coagulation, allograft rejection, complement, epithelial-mesenchymal transition, and xenobiotic metabolism. Conversely, the majority of the genes which differentially expressed in the low-risk cohort were concentrated in pathways connected to protein secretion, fatty acid metabolism, heme metabolism, oxidative phosphorylation, and androgen response. These insights imply that the poor prognosis in KIRC may be linked to the activation of genes involved in the coagulation pathway, whereas the upregulation of genes associated with the protein secretion pathway could potentially improve the prognosis of KIRC patients. Furthermore, according to the GO enrichment analysis, genes that showed variations in expression between 2 risk categories were primarily enriched in pathways for example antigen binding, the immunoglobulin complex, and leukocyte-mediated immune response** (Figure 5A, B)**. KEGG enrichment analysis emphasized the notable concentration of genes with distinct expression patterns among 2 different risk cohorts in leukocyte-mediated immune response and immunoglobulin-mediated immune response pathways **(Figure 5C, D)**. These results from the enrichment analysis suggest that the different prognosis between 2 different risk cohorts maybe due to dis-regulation of leukocyte and their immunogobulin secretion activity.

### Tumor mutation burden analysis

**Figure 6:**
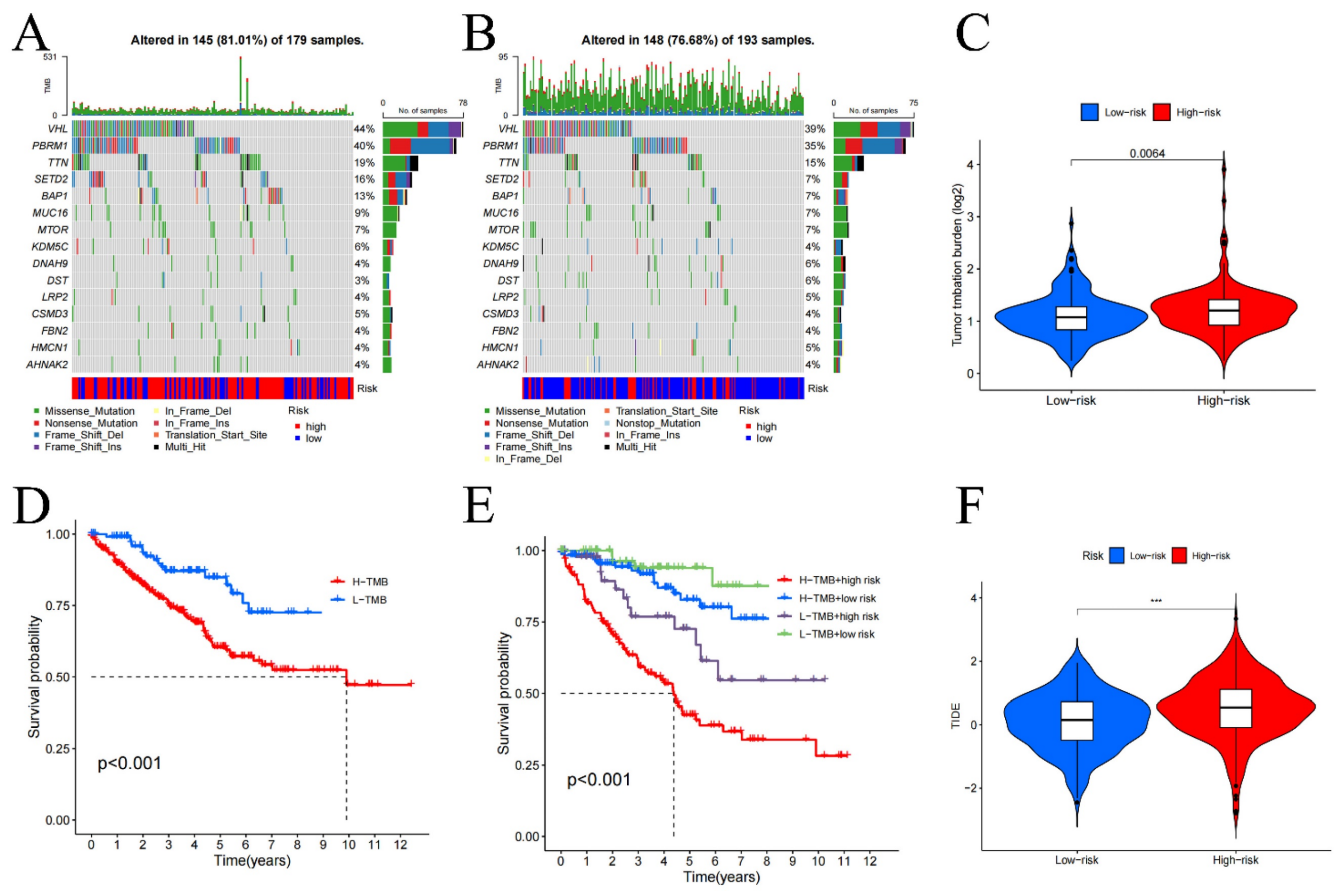
Tumor mutation burden (TMB) in high **(A)** and low-risk groups **(B)**, differential analysis of TMB between high and low-risk groups **(C)**, and survival curves **(D-E)**. TIDE analysis of immune evasion and immunotherapy (F). *p < 0.05, **p < 0.01, ***p < 0.001.

We performed a statistical analysis of the TMB and used waterfall charts **(Figure 6A, B)** to display the mutation patterns of the 2 risk categories' samples. Comparing the 2 risk cohorts, TMB is substantially higher for high-risk category (p-value=0.0064) **(Figure 6C)**. In high-risk samples, the genes VHL (44%), PBRM1 (40%), TTN (19%), SETD2 (16%), and BAP1 (13%) exhibited the highest mutation rates. While the most altered genes in the low-risk group were VHL (39%), PBRM1 (35%), and TTN (15%). Additionally, survival analysis comparing low TMB group and high TMB group **(Figure 6D, E)** demonstrated that in contrast to the group with low TMB, the OS was considerably worse in high TMB group (p<0.001). These results point to a noteworthy connection between genes mutation numbers and the prognosis of KIRC patients, indicating that a higher TMB correlates with poorer prognosis.

### Tumor microenvironment and immune-related analysis

In our investigation of the TME, we utilized violin plots **(Figure S9A)** to demonstrate how the 2 risk categories' cell infiltration differs from one another. The research's findings demonstrated that comparing to low-risk cohort, high-risk cohort has higher stromal cell, and immune cell scores (all p-values<0.01). This suggests a notably greater presence of immune and stromal cells within the samples from the high-risk group. Further, immune phenotyping analysis highlighted significant differences in the immune characteristics between the two risk categories** (Figure S9B)** (p-value=0.001), emphasizing the distinct immune landscapes that correlate with patient risk categorization. Immune function analysis revealed that functions such as cell-mediated immunity, pro-inflammatory response, macrophage phagocytosis, co-stimulation of immune cells, and enhanced activity of helper T cells were noticeably greater in the group at high risk (all p-values<0.01), as shown in **Figure S9C**. In contrast, increased mast cell activity and a secondary tumor necrosis factor response, both significantly correlated with improved prognosis, were more prevalent in low-risk cohort (all p-value<0.01). Additionally, the analysis of immune cell infiltration in tumor tissues **(Figure S9D)** indicated that when compared to low-risk samples, the prevalence of regulatory T cells, plasma cells, follicular helper T cells, and M0 macrophages was considerably higher in high-risk samples (all p-value<0.01). On the other hand, the presence of monocytes, M1 macrophages, resting dendritic cells, resting NK cells, and mast cells was notably higher in low-risk samples (all p-value<0.01). These results were further corroborated by the risk score and immune cell infiltration association analysis, as detailed in **Figure S10**, underscoring the significant variances in immune profiles between the two risk groups. The analysis specifically highlighted that the infiltration of regulatory T cells increases markedly with higher risk scores, suggesting their potential role in contributing to the adverse prognosis of KIRC. It is noteworthy, although CD4+ T cells appear to act as a protective factor, as their numbers increase with decreasing risk scores, there was no discernible variation in CD4+ T cell infiltration between 2 risk categories. Additionally, in comparison to low-risk cohort, the high-risk cohort had higher TIDE scores **(Figure 6F)**, suggesting that immunotherapy may work better for people in the low-risk category.

### Drug sensitivity prediction

Following an assessment of 198 anti-cancer medications' sensitivity in high- and low-risk categories, we employed box plots to illustrate significant differences in response to 80 of these drugs between the two groups** (Tables S6-S8)**. Specifically, the high-risk group generally showed heightened sensitivity to drugs that target the PI3K/AKT signaling pathway **(Figure S11K)**, including Taselisib, Buparlisib, Afuresertib, AZD8055, Taselisib, Pictilisib, GNE-317, Alpelisib, AZD8186, MK-2206, AZD2014, and PF-4708671, **Figure S11A-J** exhibit some of the drugs. Samples in low-risk groups are more sensitive to drugs that block EGFR signaling pathway** (Figure S11K)**, including AZD3759, Osimertinib, Gefitinib, Erlotinib and Afatinib.

### Experimental validation *in vitro*

The Human Protein Atlas (HPA) database's immunohistochemical staining pictures were utilized to compare the protein expression levels of each FIGs in KIRC tissues to those in normal kidney tissues, as shown in **Figure S10A**. RT-qPCR analysis **(Figure S12B-C)** further quantified expression differences, revealing that the expression of HLA-DRA and CLDN4 was much higher in normal tissues than in cancerous tissues. In contrast, compared to normal tissues, tumor tissues showed noticeably elevated expression levels of SAA1, MDK, and ISG15. LTF showed increased expression in Caki-1 cells compared to normal tissues, despite this, there was no discernible variation in its expression between Caki-2 cells and normal tissues.

## Discussion

RCC, especially its subtype KIRC, is marked by a high mortality rate of 30%-40% and an elusive pathogenesis 1. Traditional TNM staging has shown limited effectiveness in prognosis prediction 2, highlighting the need for innovative prognostic models. Recent research has underscored the significant role of fibroblasts within the tumor microenvironment in driving cancer progression 16. Leveraging single-cell data, we developed a risk score that aims to improve prognostic predictions for patients with KIRC. The risk score that utilized 6 FIGs is an independent predictive factor. It was discovered that patients placed in the high-risk category had a less favorable outcome. A nomogram, generated using clinical data and a risk score, demonstrated robust predictive accuracy. Additionally, enrichment analysis exhibited that genes linked to the protein secretion pathway might contribute to a more favorable prognosis. Through drug sensitivity analysis, this research also provides a crucial tool for tailoring anti-cancer treatment strategies in KIRC patients.

TNF expression in fibroblasts may play a significant role in KIRC. TNF, mainly produced by activated monocytes/macrophages 17, can kill and inhibit tumor cells 18 and promote the phagocytosis 19. Our PPI demonstrated the core role of TNF in FIGs. Therefore, we suspect that TNF in fibroblasts can be a research direction for understanding KIRC. The risk heatmap revealed that CLDN4, LTF, and HLA-DRA are low-risk genes, whereas SAA1, MDK, and ISG15 were classified as high-risk genes. CLDN4, Claudin-4 mainly responsible for tight connections between cells 20. The overexpression of CLDN4 may inhibit epithelial-mesenchymal transition 21. LTF, lactoferrin can enhance the body's immune response by maintaining the balance of iron ions 22. HLA-DRA encodes α chain in Class II HLA molecules which plays a significant apart in human immune response (especially allograft rejection) by being presented on antigen-presenting cells, particularly dendritic cells 23 and macrophages 24. SAA1, Serum Amyloid A1 is an acute phase protein secreted by liver cells under the regulation of pro-inflammatory cytokines 26. It has been recognized overexpression in multiple cancers, including lung and breast cancer 27. MDK, midkine, serves as a vital pluripotent cytokine in the growth and development of the nervous system 28. MDK promotes the occurrence and development of various tumors by activating signaling pathways like PI3K/AKT 29. ISG15, Interferon-stimulated gene 15, is a gene encodes a specific protein which can interacts with cell cycle proteins, promote the progression of the cell cycle 30. GSEA result can also offer clues of the mechanism for FIGs to KIRC. Active coagulation pathways may promote cancer growth and metastasis. Thrombin, as a core member of coagulation, can stimulate platelet release of TGFβ1 which can accelerate cancer cell proliferation and metastasis 31. Thrombin also can trigger a series of intracellular signal transduction, including the PI3K/AKT pathway, enhancing the cell's proliferation, migration, and invasions 32. Complements can promote tumor cell migration (loss of adhesion molecules) by combing with integrins which is on tumor cells surface 33. Epithelial-mesenchymal transition may be mediated by overexpression of CLDN4, is considered a risk factor for KIRC, for its aiding cancer metastasis 34. Heme metabolism is considered a protective factor, it may be due to heme can reduce the angiogenesis of tumor environment which reduce the metastasis 35. Oxidative phosphorylation can inhibit the aerobic glycolysis 36 which can inhibit cancer development. Protein secretion can enhance the immunity which help the body to fight tumors 37. We have down TME analysis which helps us finding out the unknown of KIRC patients' TME. Stromal cells are higher in high-risk group, it may be caused by the elevated tumor-stromal cell interaction which can promote tumor growth and angiogenesis. Regulatory T cells are responsible for calming hyper-activated immune response down, avoiding excessive immune response damage to the body. Their large infiltration in high-risk samples might indicate poor prognosis, which make them become a potential target for immunotherapy, CD4+ T cells can play apart in better prognosis for its number raise with the decrease of risk score. This information provides clues for future immunotherapy targets.

Drug sensitivity analysis has identified two key pathways: the PI3K/AKT and EGFR signaling pathways, both of which are essential in controlling cellular functions including growth, survival, and differentiation 38. These pathways represent potential focal points for developing personalized treatments for KIRC. It is hypothesized that stromal cells, particularly fibroblasts, may secrete TGFβ. This protein could interact with receptor tyrosine kinases on tumor cells, thereby activating the PI3K/AKT pathway, which is believed to be upregulated in patients from high-risk group, suggesting a target for therapeutic intervention.

Our study offers several advantages. Notably, we developed a nomogram based on FIGs that has not been previously reported and successfully used it to predict survival time in KIRC patients, setting it apart from other studies. For comparison, the prognostic model for KIRC developed by Wang, C., *et al.* (2023) 2 also utilized transcriptomic data and single-cell multi-omics information and showed predictive utility for KIRC. However, their study did not incorporate TMB, nor did it explore the selection of anti-cancer drugs, we also summarized the classification of anti-cancer drugs that we identified vital for high- and low-risk groups. Comparing to the KIRC model creating by Muhammad Khan *et al.* (2022) 10 using pyroptosis related genes, we used RT-qPCR to validate the expression of the core genes used to construct risk score, while they did not. In contrast to the cuproptosis-related gene model by Liu, Y., *et al.* (2023) 4, our risk score was validated more extensively across all clinical subgroups, leading to more robust findings. Moreover, our risk score demonstrated a higher accuracy with an AUC of 0.744 compared to the telomere-related gene risk prognostic model by Li, S. C., *et al.* (2022) 39, which had an AUC of 0.721. Despite these strengths, it is critical to recognize the constraints on our research. The data utilized were sourced solely from a single database and have not been validated with external datasets. Consequently, additional clinical experiments are required to further substantiation.

## Conclusions

The risk score and accompanying nomogram effectively predict outcomes for KIRC patients. Raised TMB and an active coagulation pathway may be linked to a worse outcome in high-risk group. According to drug sensitivity research, medications that target the PI3K/AKT signaling pathway are effective for patients in high-risk category, whereas those in the low-risk category show greater sensitivity to inhibitors of the EGFR pathway. Nonetheless, these findings need to be confirmed through additional experimental and clinical studies.

## Supplementary Material

Supplementary figures and tables.

## Figures and Tables

**Table 1:** Clinical information of the patients in the test and train groups.

Characteristics	Train cohort (n=267)		Test cohort (n=266)		Entire cohort (n=533)	
	n	%	n	%	n	%
Age						
≤65	166	62.17	183	68.8	349	65.48
>65	101	37.83	83	31.2	184	34.52
Status						
Alive	177	66.29	181	68.05	358	67.17
Dead	90	33.71	85	31.95	175	32.83
Gender						
Female	94	35.21	94	35.34	188	35.27
Male	173	64.79	172	64.66	345	64.73
Stage						
Stage I	137	51.31	130	48.87	269	50.09
Stage II	25	9.36	32	12.03	57	10.69
Stage III	63	23.60	60	22.56	123	23.08
Stage IV	41	15.36	42	15.79	83	15.57
Unknow	1	0.37	2	0.75	3	0.56
T stage						
T1	140	52.44	133	50.00	273	51.22
T2	34	12.73	35	13.16	69	12.95
T3	88	32.96	92	34.57	180	33.77
T4	5	1.87	6	2.26	11	2.06
M stage						
M0	212	79.40	210	78.95	422	79.17
M1	38	14.23	41	15.41	79	14.82
Unknow	17	6.37	15	5.64	32	6.01
N stage						
N0	120	44.94	120	45.11	240	45.03
N1	8	3.00	8	3.01	16	3.00
Unknow	139	52.06	138	51.88	277	51.97
Race						
White	232	86.89	230	85.19	462	86.68
Black or African American	29	10.86	27	10.00	56	10.51
Asian	2	0.74	6	2.22	8	1.50
Unknow	4	1.50	3	2.59	7	1.31

**Abbreviation:** M stage: metastasis stage; N stage: Node stage; T stage: Tumor stage.

**Table 2:** Clinical information for 533 patients in different risk categories.

Characteristics	High-risk group (n=264)		Low-risk group (n=269)	
	n	%	n	%
Age				
≤65	170	64.39	179	66.54
>65	94	35.61	90	33.46
Status				
Alive	136	51.52	222	82.53
Dead	128	48.48	47	17.47
Gender				
Female	87	32.95	101	37.55
Male	177	67.05	168	62.45
Stage				
Stage I	94	35.61	173	64.31
Stage II	24	9.09	33	12.27
Stage III	78	29.55	45	23.08
Stage IV	67	25.38	16	5.95
Unknow	1	0.38	2	0.74
T stage				
T1	99	37.50	174	64.68
T2	33	12.50	36	13.38
T3	121	45.83	59	21.93
T4	11	4.17	0	0
M stage				
M0	181	68.56	241	89.59
M1	63	23.86	16	5.95
Unknow	20	7.58	12	4.46
N stage				
N0	114	43.18	126	46.84
N1	12	4.55	4	1.49
Unknow	138	52.27	139	51.67
Race				
White	230	87.12	232	84.98
Black or African American	29	10.98	27	9.89
Asian	2	0.76	6	2.20
Unknow	3	1.14	4	2.93

**Abbreviation:** M stage: metastasis stage; N stage: Node stage; T stage: Tumor stage.

## Abbreviations

AUC: area under the curve
CI: Confidence Interval
EGFR: epithelial growth factor receptor
FIG: fibroblast immune-related genes
GSEA: Gene set enrichment analysis
HIF: Hypoxia Inducible Factor
HR: Hazard ratios
KEGG: Kyoto Encyclopedia of Genes and Genomes
KIRC: renal clear cell carcinoma
K-M: Kaplan-Meier
K-SFM: Keratinocyte SFM
LASSO: Least absolute shrinkage and selection operator
LncRNA: Long non-coding RNA
OS: Overall survival
PCA: Principal component analysis
PFS: progression free survival
PPI: protein-protein interaction
RCC: renal cell carcinoma
ROC: Receiver operating characteristic
ssGSEA: Single-Sample Gene Set Enrichment Analysis
TCGA: The Cancer Genome Atlas
TGF: transforming growth factor
TIDE: Tumor immune dysfunction and exclusion
TISCH: Tumor Immuno-Single Cell Hub
TMB: Tumor Mutation Burden
TME: Tumor microenvironment
TNF: tumor necrosis factor
