# Supplementary material for: Identification and Validation of a Prognostic Signature Based on Fibroblast Immune-related Genes to Predict the Prognosis and Therapeutic Response of renal clear cell carcinoma by Integrated Analysis of Single-Cell and Bulk RNA Sequencing Data
Source: J Cancer. 2024 Sep 23;15(18):5942–55. doi: 10.7150/jca.100194 (PMC11493018; doi:10.7150/jca.100194)
Supplement: Supplementary file 1 — Supplementary figures and tables. [file jcav15p5942s1.pdf]

Supplementary Data

Figure S1 Heatmaps of the expression of 6 FIGs (A-C). Risk score curves for each patient's survival status (D-F) and scatter plots (G-I).

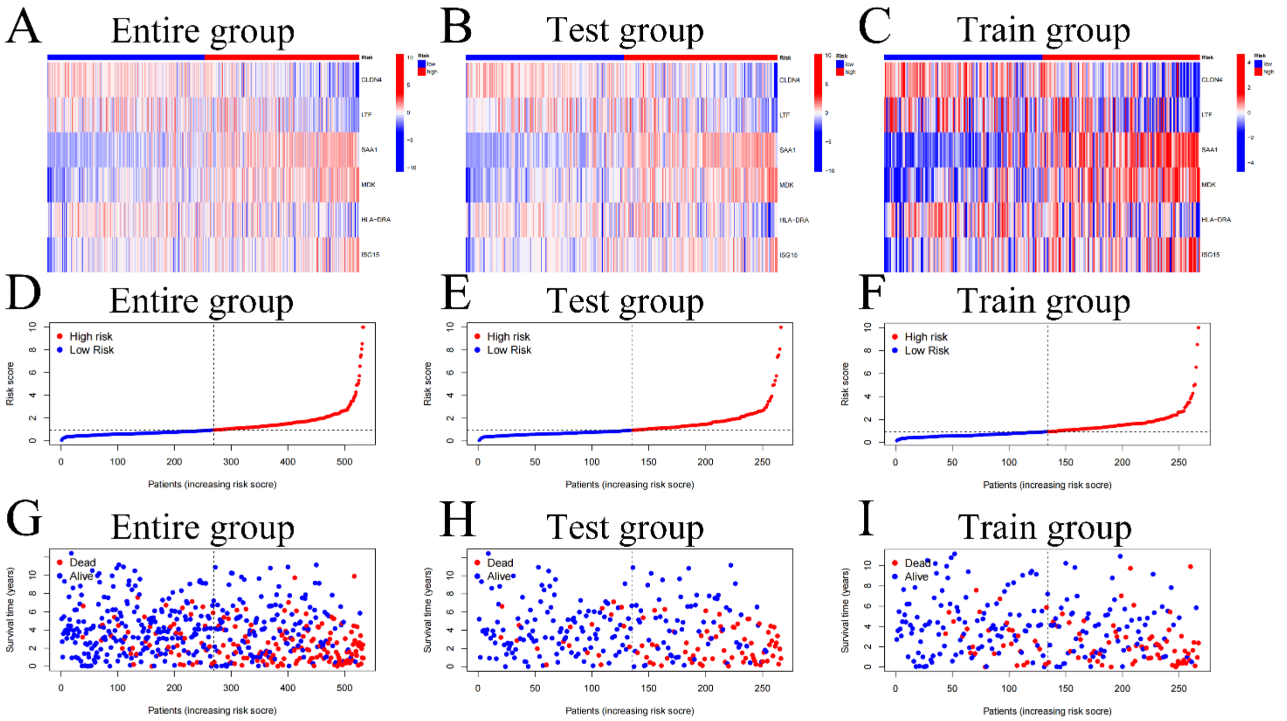

Figure S2 Relationship between risk genes and model risk scores (A-L).

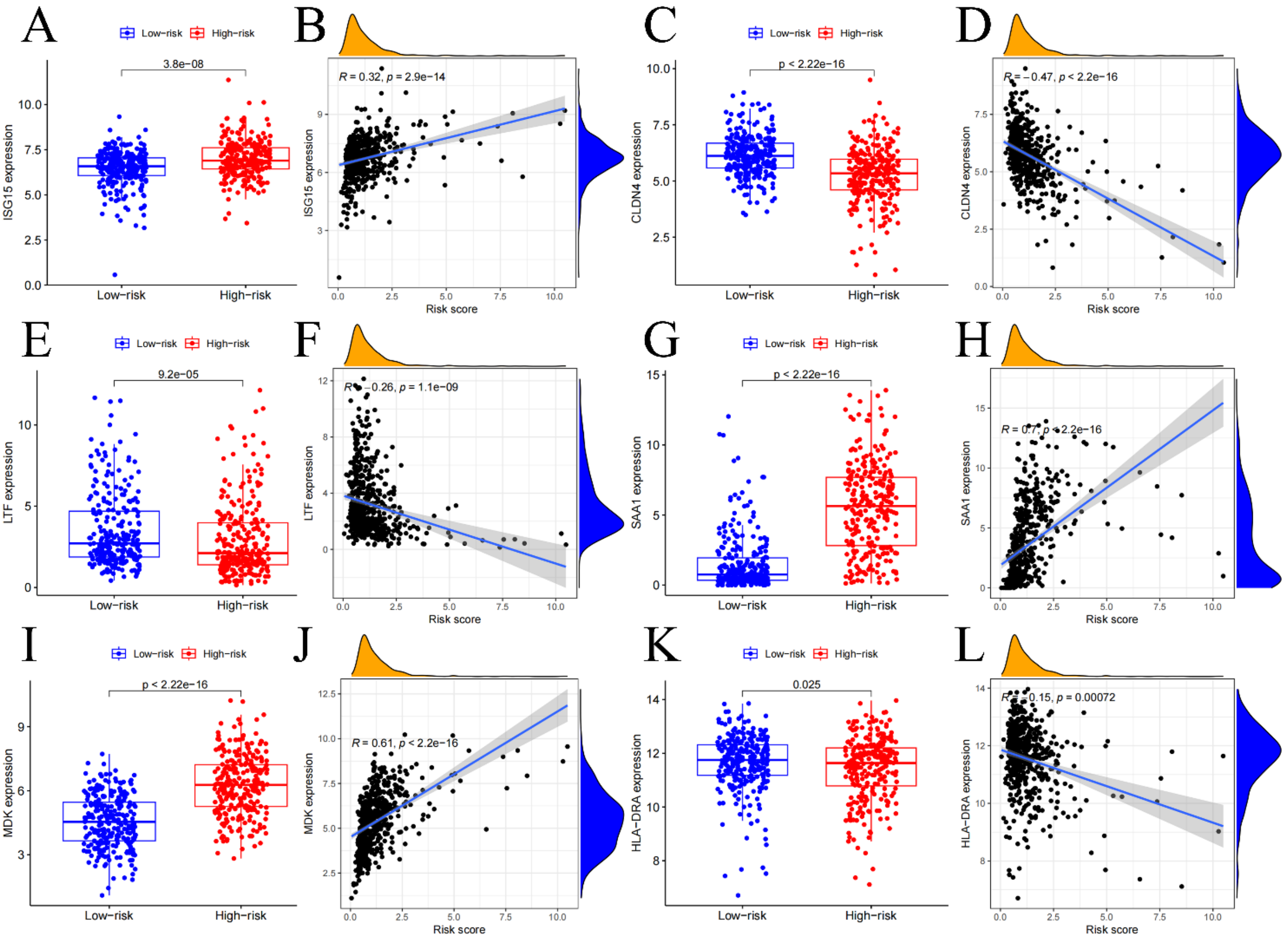

Figure S3 Survival curves for risk genes (A-F).

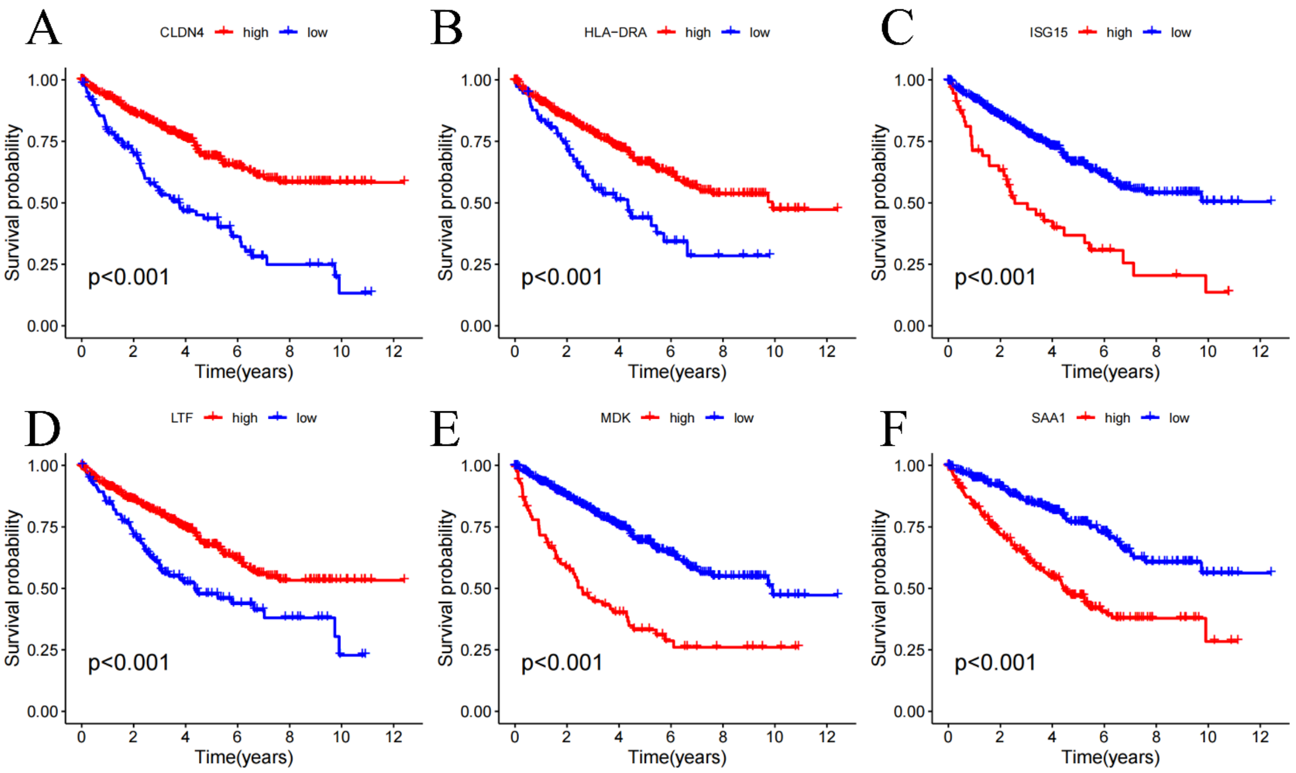

Figure S4 Univariate (A) and multivariate (B) Cox analyses of age, gender, stage, grade, and risk score showed that age, stage, grade, and risk score are independent prognostic factors. Clinical relevance circle diagram (C) and risk heatmap (D).

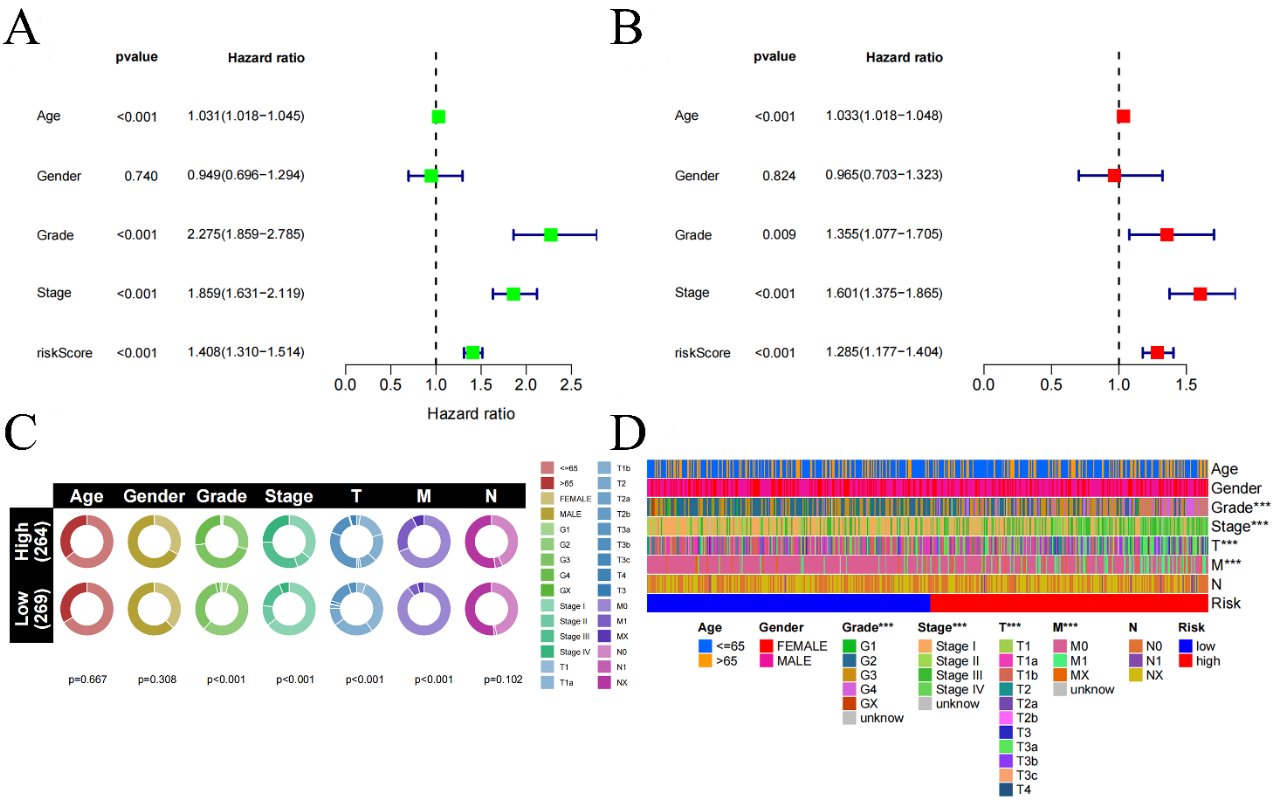

Figure S5 Scatter plots of risk scores in different clinical groupings (A-G), PCA principal component analysis of all fibroblast immune-related genes (H) and model genes (I).

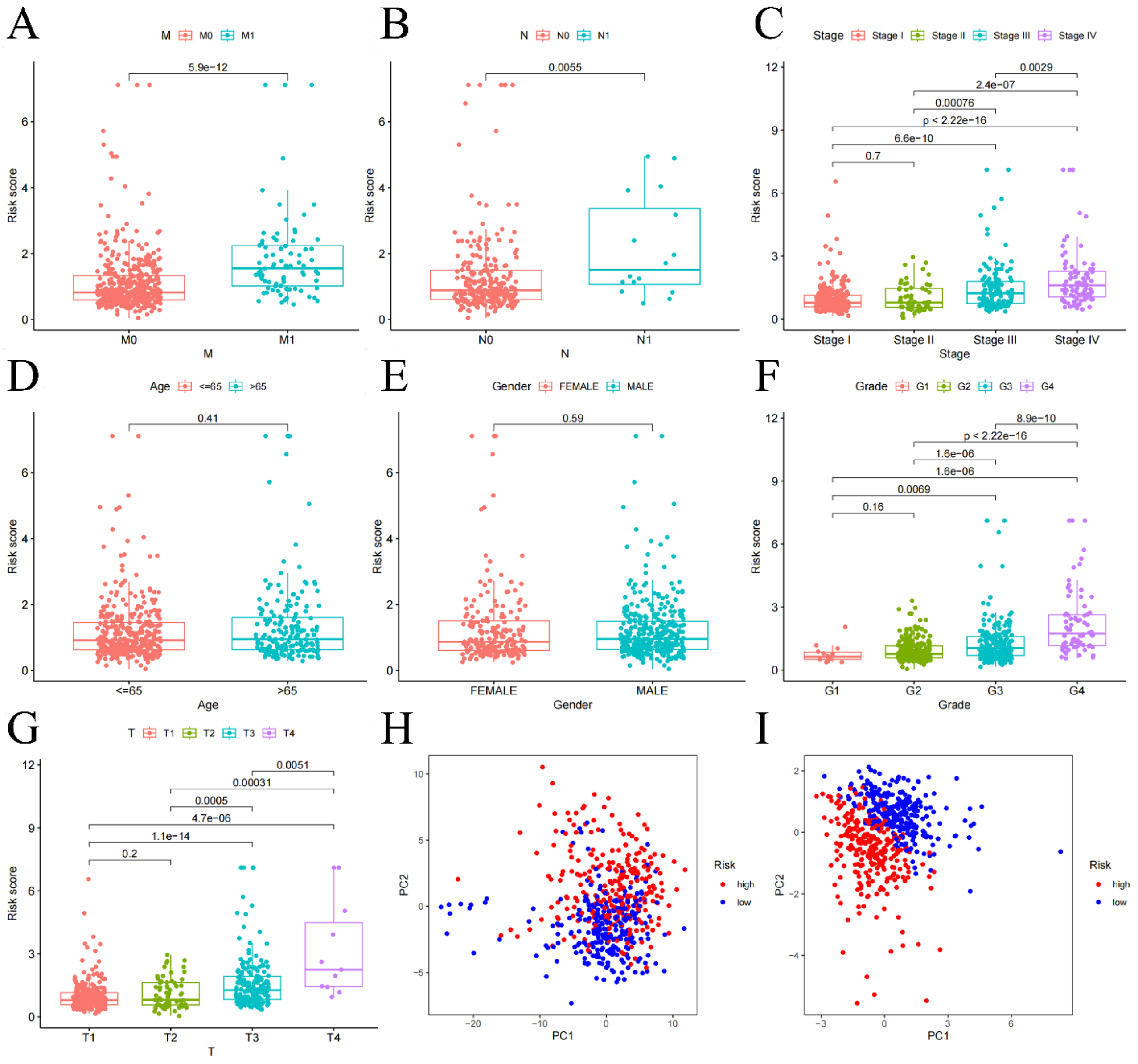

Figure S6 Clinical grouping validation of the risk model (p<0.01) (A-H).

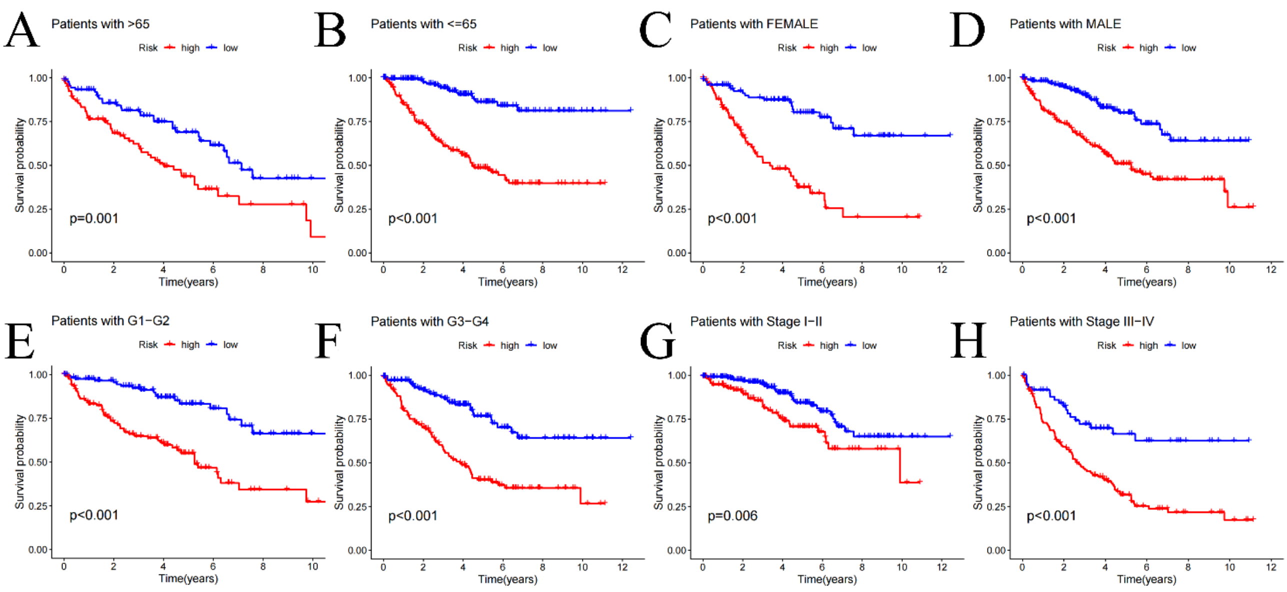

Figure S7 Survival curves for clinical subgroups (A-G).

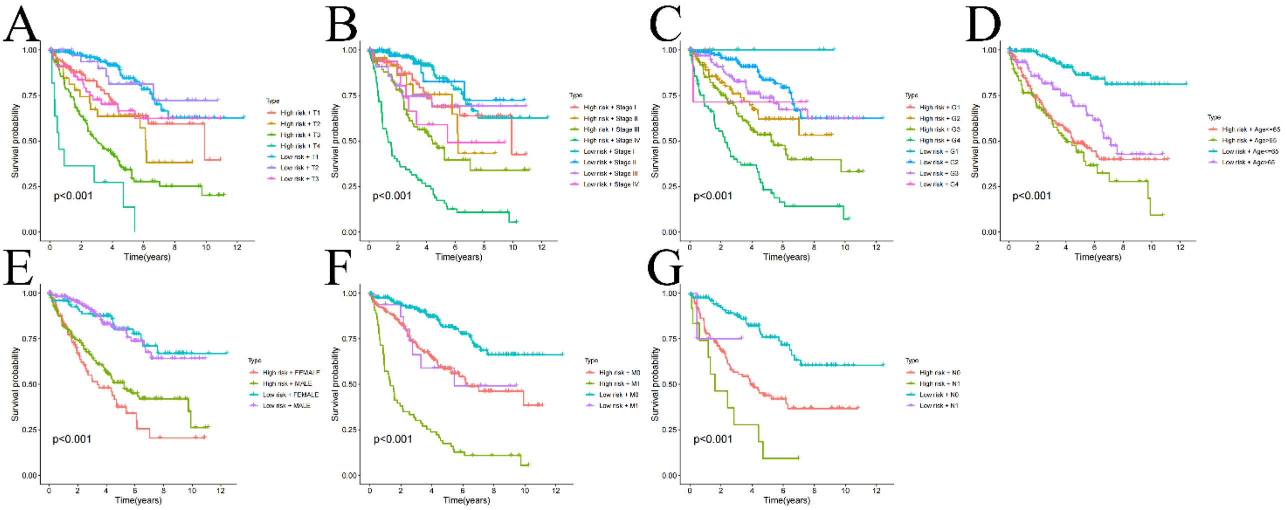

Figure S8 Results of GSEA enrichment analysis performed with 6 genes and files, showing the top 5 enriched pathways in high and low-risk groups (A-L).

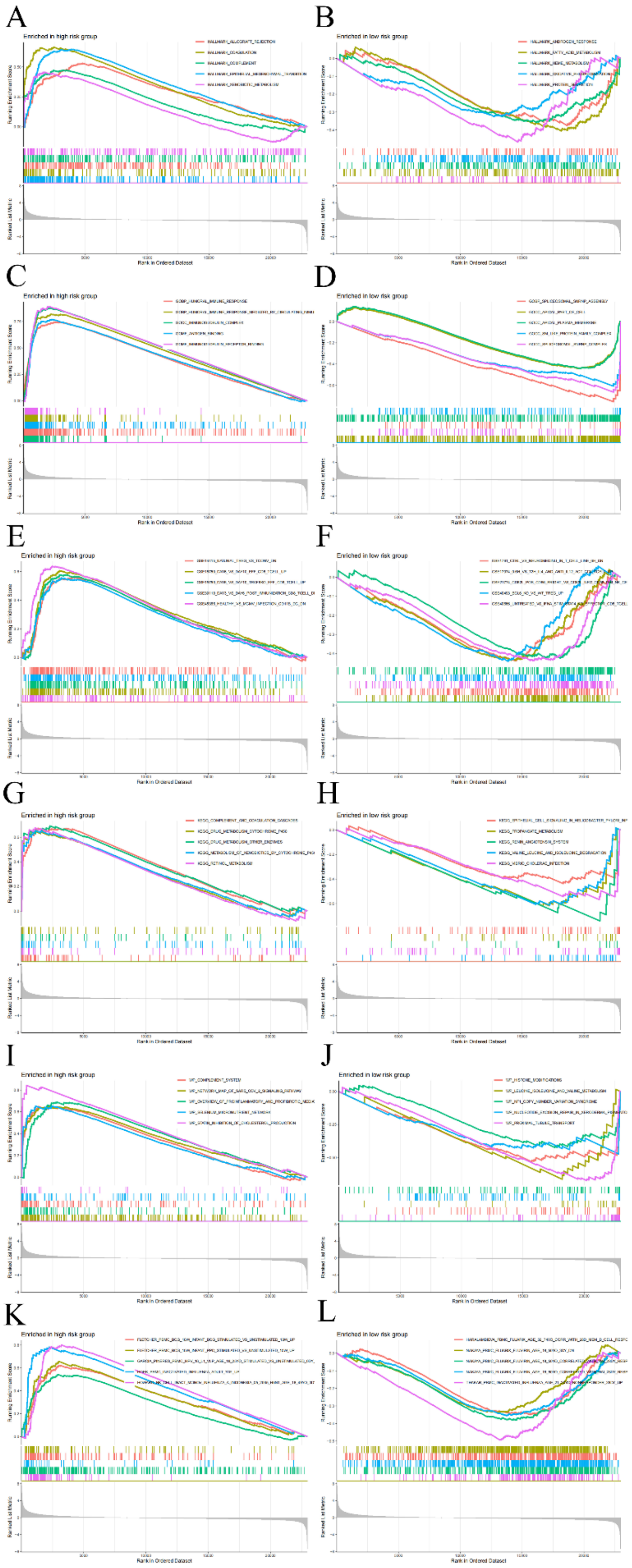

Figure S9 Tumor microenvironment (TME) analysis (A) showing the infiltration of stromal and immune cells in high and low-risk groups, immune typing (B), tumor tissue immune cells (C), and immune function analysis (D).

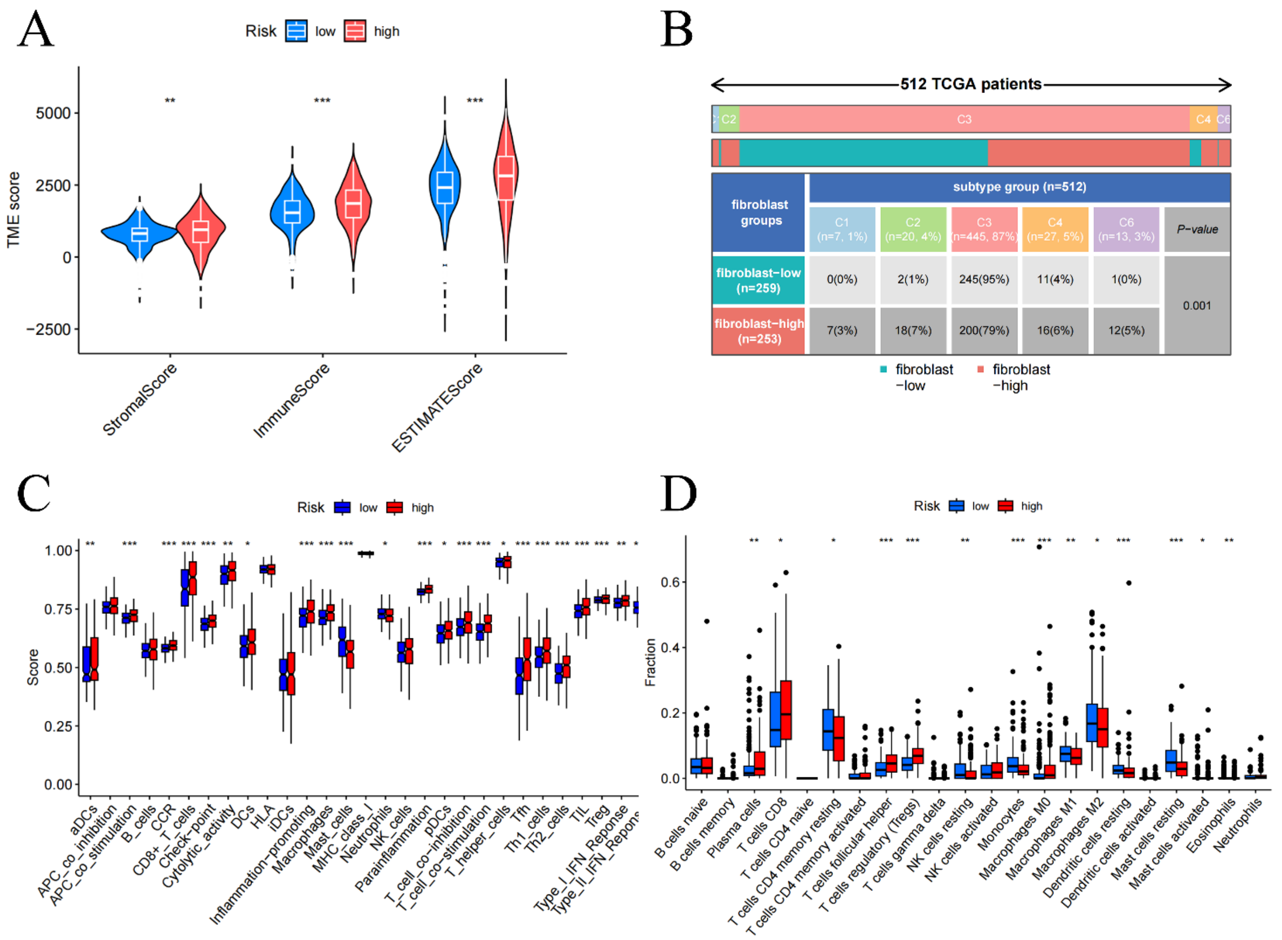

Figure S10 Correlation of immune cells with risk score (A-N).

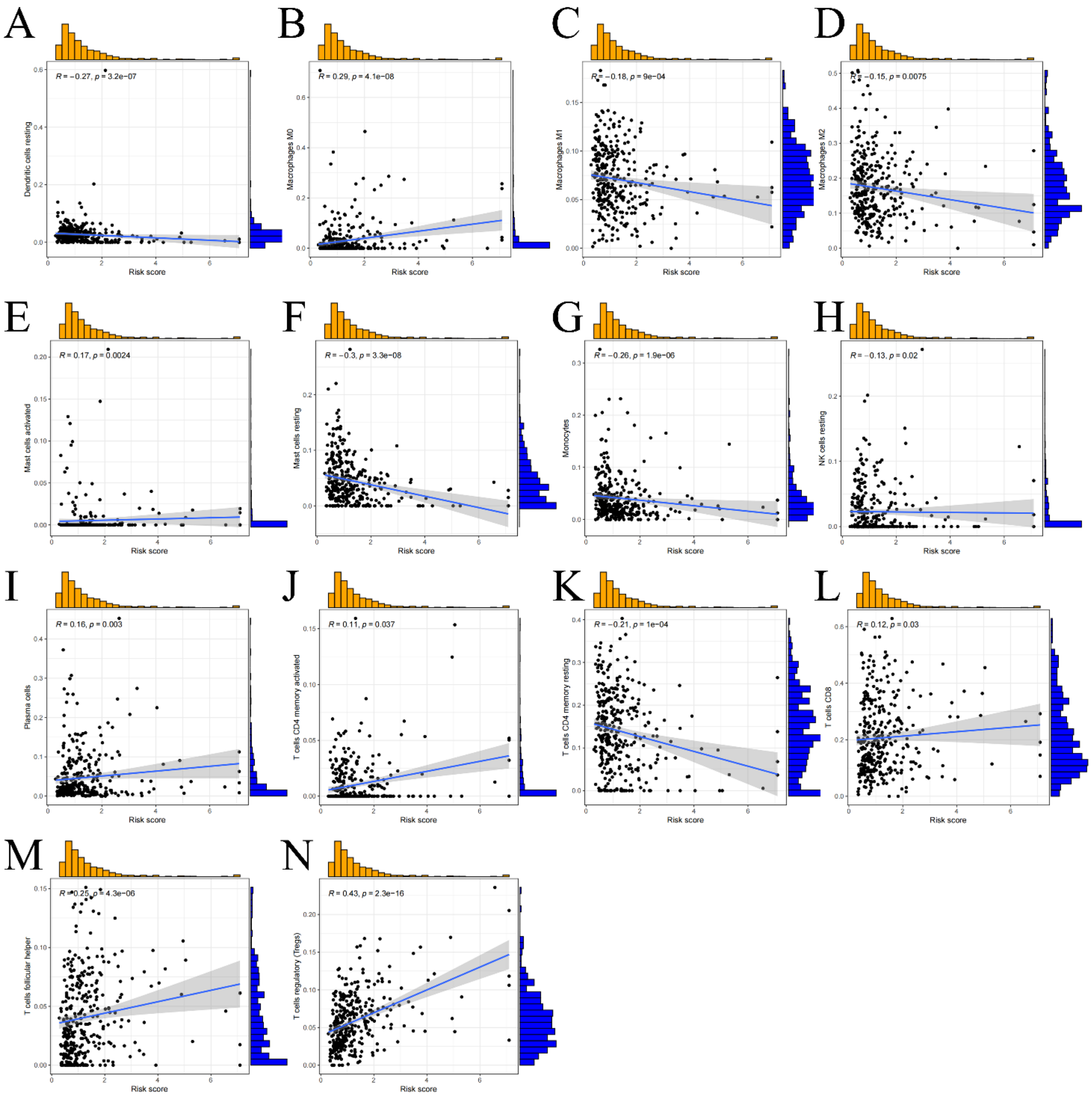

Figure S11 Differential analysis results of drug sensitivity between high and low-risk groups (L), showing that the high-risk group is sensitive to some PI3K/AKT drugs (A-K).

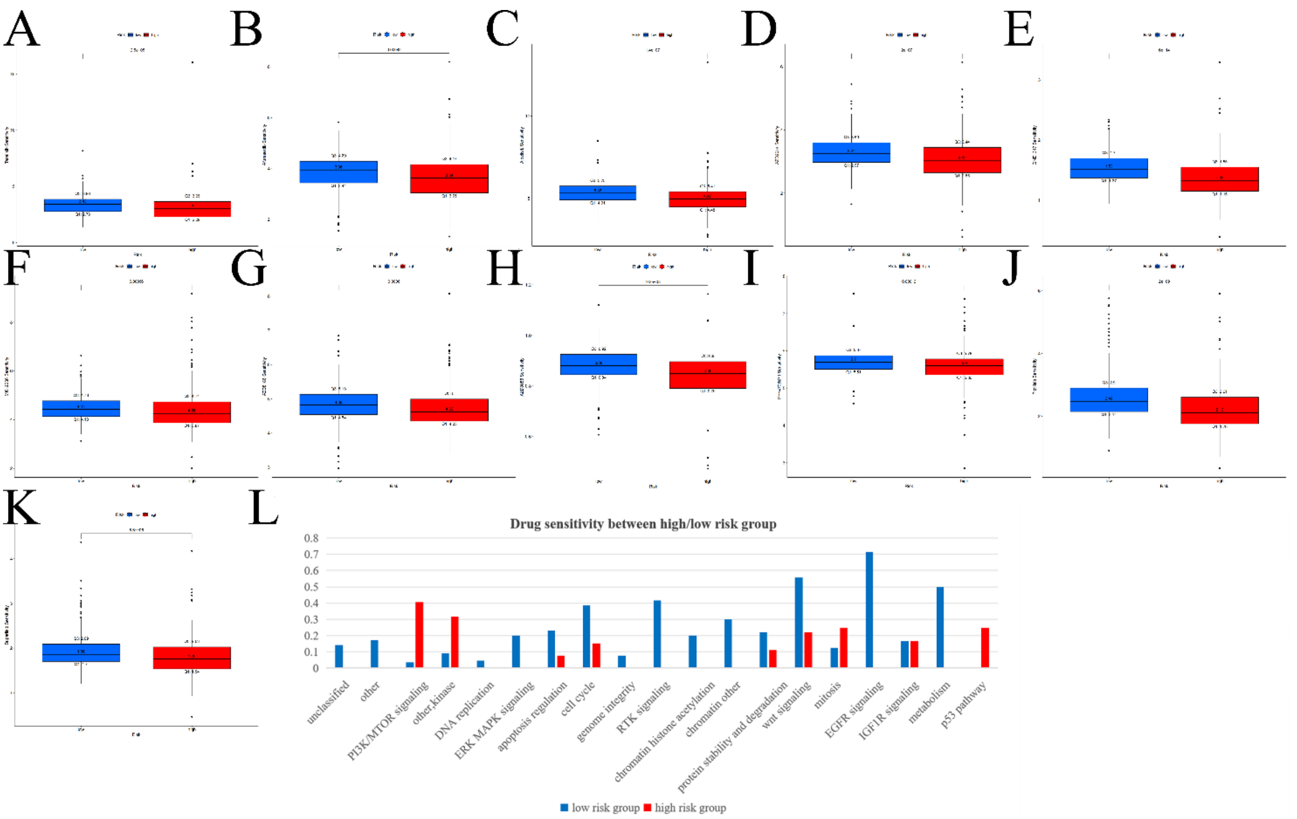

Figure S12 Experimental validation of the risk model in vitro included immunohistochemical staining images of select FIGs proteins in KIRC and normal tissues (A), as well as the relative expression of 6 FIGs in various cell lines (B-C). *p < 0.05, **p < 0.01, ***p < 0.001.

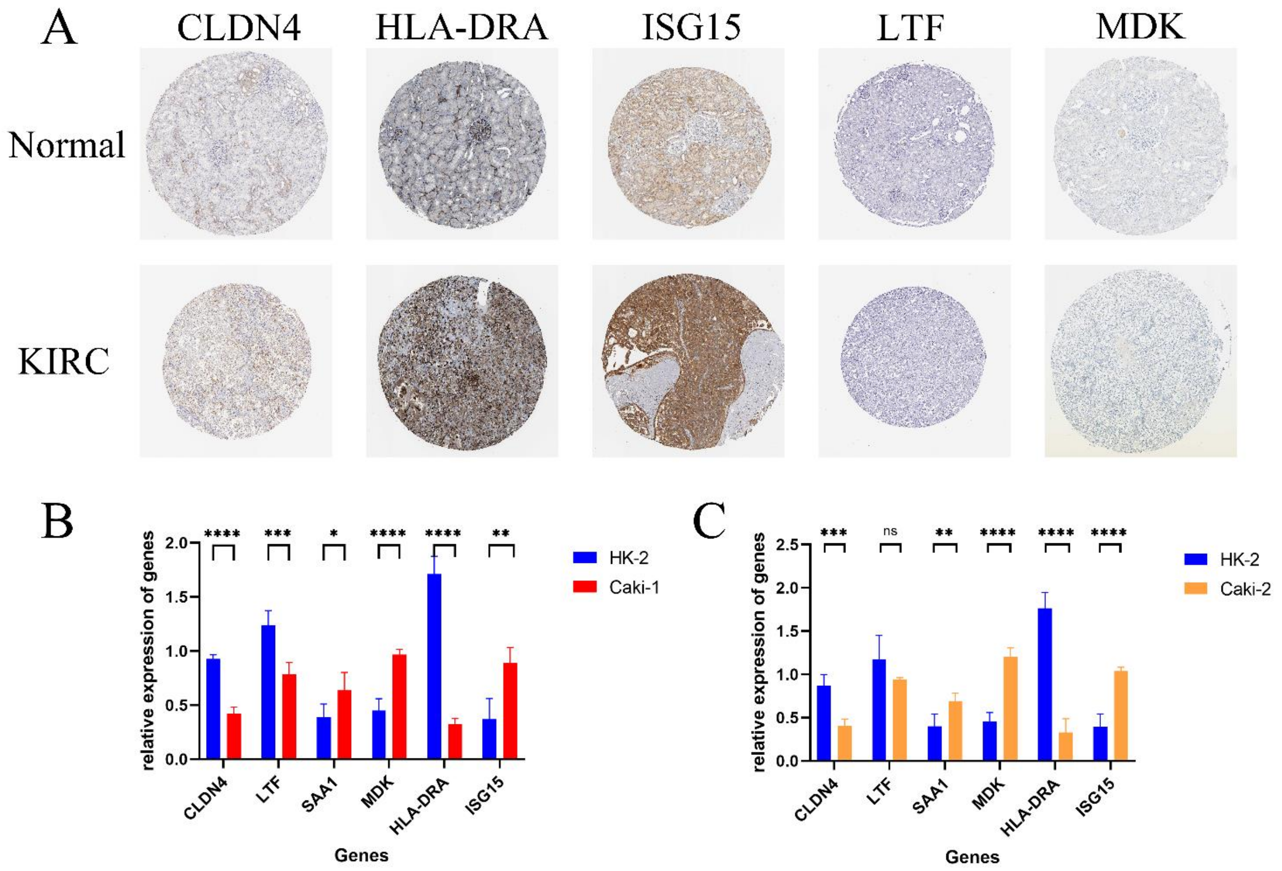

Table S1 Primer sequences for 6 fibroblast immune-related genes (5'→3').

Gene Id	Primer F	Primer R
CLDN4	CTTTGCTGCAACTGTCCACC	CCTACCCGGAACAGAGGAGA
LTF	AACCACCATGGGTGAAGGTC	TCCTTGGACCAACAGCATCC
SAA1	GACAGTCAGGAGGGAGACGA	TACCCTCTCCCCGCTTTGTA
MDK	GAAGAAGGCGCGCTACAATG	CCCTTCTCCTTTCGCTGACC
HLA-DRA	CTCACAAACAGCCCTGTGGA	ACATTTCCTTTCCCCACCCTG
ISG15	GGCCAGGTTCTAAGTGTGCT	GCGTCACACAGGTTCAGAGA

Table S2 Firboblast cell differentially expressed genes.

Gene	log2FC	Percentage (%)	Adjusted p-value
NDUFA4L2	3.32	99.1	0
CRYAB	3.19	99.7	0
FXYD2	2.79	98.9	0
RARRES2	2.76	98	0
CD24	2.74	99.1	0
RHEX	2.71	98.6	0
NNMT	2.48	99.5	0
MGST1	2.38	97.4	0
CXCL14	2.38	96.5	0
CLU	2.37	97.3	0
ANGPTL4	2.32	99.1	0
KRT19	2.31	97.9	0
KRT18	2.3	99.2	0
DEPP1	2.17	96.2	0
SERPINE1	2.14	95.5	0
IGFBP3	2.1	93.5	0
GPX3	2.09	98.3	0
PDZK1IP1	2.02	96.1	0
HSPB8	1.96	98.2	0
KRT8	1.95	98.3	0
UGT2B7	1.87	98.5	0
SLC17A3	1.82	91.7	0
BNIP3	1.81	97.3	0
FABP7	1.77	95.5	0
CAV1	1.76	96.4	0
ADIRF	1.72	96.4	0
IGFBP7	1.72	96.7	0
UCHL1	1.72	95	0
C1S	1.7	98.5	0
C19orf33	1.64	96.2	0
S100A1	1.63	97.4	0
CYB5A	1.57	96.8	0
RRAD	1.53	95.2	0
HILPDA	1.52	96.8	0
ANXA4	1.5	97.1	0
CA12	1.47	97.3	0
IGFBP5	1.43	91.4	0
PLOD2	1.42	97.1	0
RBP5	1.42	96.4	0
ERRFI1	1.42	96.5	0
PGRMC1	1.42	96.5	0
BEX3	1.42	96.4	0
S100A13	1.4	97	0
CLDN4	1.39	92.9	0
TNFRSF12A	1.39	93.5	0

CP	1.38	96.2	0
EGLN3	1.36	96.8	0
CCDC146	1.35	95.6	0
MT3	1.35	92	0
MET	1.35	96.7	0
BHMT2	1.32	97.4	0
PAX8	1.3	97.4	0
VCAM1	1.28	97.1	0
ENO2	1.25	96.7	0
AKR1C3	1.24	94.2	0
PPP1R1A	1.23	94.8	0
CALD1	1.23	97.4	0
DEFB1	1.23	91.5	0
LTF	1.23	80.8	0
CAV2	1.22	95.3	0
LINC01320	1.19	96.2	0
VEGFA	1.18	96.5	0
TFPI	1.18	95.9	0
NDRG1	1.16	97.6	0
PDLIM1	1.14	96.8	0
PFKP	1.12	97.7	0
WFDC2	1.12	90.6	0
SLC22A5	1.11	94.7	0
BHMT	1.11	90.2	0
ABCC3	1.11	97	0
FABP6	1.11	93.8	0
NOL3	1.08	96.2	0
CAVIN3	1.06	95.2	0
ATP1B1	1.06	97.4	0
LOX	1.04	93.8	0
CYP3A5	1.04	94.7	0
GNG11	1.03	90.6	0
C1R	1.03	95.9	0
ERGIC1	1.02	97.1	0
TPM1	1.02	96.5	0
YBX3	1	97.9	0
RTEL1-TNFRSF6B	1	89.7	0
HPN	1	93	0
CISD1	1	95.2	0
PLEKHA5	0.99	97.1	0
LINC02532	0.99	94.1	0
TCEA3	0.99	93.8	0
AK4	0.97	96.4	0
ITGA3	0.97	92.6	0
CDH6	0.97	94.2	0
SPARC	0.97	92.1	0
TM4SF18	0.96	92.3	0
GADD45A	0.96	95.8	0

S100A16	0.95	92.3	0
ANPEP	0.93	93.6	0
AGRN	0.9	93.8	0
RNLS	0.9	91.8	0
HP	0.89	69.2	0
BACE2	0.89	93.8	0
PFN2	0.88	91.1	0
MGLL	0.87	94.5	0
CNN3	0.87	93	0
COL6A2	0.87	92.6	0
EPS8L2	0.87	97.3	0
CES2	0.86	94.1	0
SNCG	0.85	85.5	0
MAOA	0.84	92.9	0
SLC3A1	0.84	89.7	0
CRACR2B	0.84	94.2	0
NRN1	0.84	90.3	0
ABI3BP	0.82	88.5	0
CRNDE	0.81	93	0
ACSM2A	0.81	85.6	0
RND3	0.8	87.3	0
GSTA1	0.8	81.2	0
HSF4	0.78	94.7	0
GGT1	0.78	93.9	0
PDLIM4	0.78	91.7	0
C16orf74	0.78	93.6	0
CA9	0.78	93.5	0
PPP1R3C	0.78	91.2	0
MT1A	0.76	83.3	0
SPINK13	0.76	91.4	0
ITIH5	0.75	91.7	0
AOC1	0.75	89.1	0
GPX8	0.75	92.9	0
CRIP2	0.74	90.3	0
GAL3ST1	0.72	90.8	0
P4HA2	0.71	93	0
TMEM45A	0.71	89.8	0
AC245595.1	0.71	93.9	0
CDHR5	0.7	87.6	0
IL20RB	0.7	93	0
HSD3B7	0.69	91.7	0
MT1G	0.69	84.5	0
SERINC2	0.69	91.5	0
SNHG18	0.68	94.1	0
MAP1B	0.67	87.6	0
ENPP3	0.66	87	0
WWTR1	0.66	92.6	0
OSMR	0.66	93	0

PTPRK	0.66	91.7	0
MVK	0.65	92.7	0
ENPEP	0.65	91.1	0
ITGB8	0.65	88.9	0
KCNJ15	0.64	90.3	0
LAMA4	0.64	90.6	0
SERPINF2	0.64	88.2	0
REPS2	0.64	90.6	0
CFB	0.63	89.2	0
RBPMS	0.63	93.2	0
EGFR	0.62	91.2	0
ELF3	0.62	86.7	0
BCAM	0.62	89.4	0
ARHGAP29	0.62	90.5	0
CMBL	0.62	90.6	0
TSPAN12	0.61	88	0
REG1A	0.61	76.5	0
ACSM2B	0.61	83.5	0
GSTA2	0.6	79.7	0
UBD	0.6	82.1	0
GALNT14	0.59	90.9	0
BICDL2	0.59	89.4	0
LAMA5	0.59	88.5	0
CDK18	0.59	92.4	0
USH1C	0.59	89.5	0
IGKV3-11	-1.17	43.8	0
TCEAL9	0.71	94.8	2.64E-307
COL18A1	0.7	94.1	3.52E-307
TCN2	0.71	91.1	4.31E-305
SERPING1	0.99	97	3.93E-302
ASPH	0.98	95.2	9.29E-302
MIF	1.64	98.8	3.91E-300
PERP	0.93	93.6	9.73E-296
C12orf75	1.47	95.8	1.01E-295
PAM	0.83	95.2	1.13E-295
GAPDH	1.87	100	8.05E-295
CRYZ	0.94	95.2	2.37E-294
CYSTM1	1.21	95.3	6.38E-293
PON2	1.08	95.2	1.14E-292
HCFC1R1	1.1	97	3.46E-289
APP	1.01	96.2	1.21E-286
HMGN3	1.3	99.4	1.42E-281
ANO6	0.75	95.5	3.56E-281
NET1	0.69	93.8	1.54E-280
IGFBP4	1.1	92.3	1.77E-280
SAA2	2.27	86.8	1.82E-280
SPP1	1.24	99.1	7.68E-280
CD151	1.28	97.6	1.13E-279

OCIAD2	1.2	96.5	9.65E-279
SAA1	3.29	93.2	3.21E-273
ADM	1.09	95.8	1.88E-270
IGFBP6	0.73	84.4	4.97E-270
LDHA	1.66	98.6	6.70E-270
SMPDL3A	0.64	92.9	1.53E-269
TIMP1	1.26	97.3	7.54E-268
S100A10	1.58	98.8	5.92E-267
PHLDA2	0.7	87.7	7.67E-267
BIRC3	1.13	96.4	9.13E-267
RAB13	0.6	91.7	1.36E-266
TMEM176A	1.62	96.1	6.13E-265
NIT2	0.77	96.5	4.37E-264
SYTL2	0.65	91.2	1.42E-263
FN1	1.54	92.7	3.02E-262
GRAMD2B	0.61	94.8	6.04E-262
MDK	0.63	81.4	1.55E-261
RTL8A	0.63	95.5	4.93E-257
AMACR	0.63	87.4	2.25E-255
TBCA	0.97	97.9	2.51E-255
CKB	0.69	91.8	1.19E-254
LTBR	0.63	95.6	1.79E-254
NDUFC1	0.94	97.4	1.90E-251
SOD2	1.37	99.2	2.31E-249
UBR4	0.67	93.9	4.19E-249
P4HB	1.25	97.7	3.15E-246
SELENOM	1.03	93.6	3.90E-245
IFITM3	1.11	98.5	7.19E-245
UQCRQ	1.06	97.9	2.11E-244
SEC61G	1.29	97.1	3.53E-243
KHK	0.62	87.6	3.95E-243
RTL8C	0.72	96.8	4.01E-243
AIG1	0.64	95.2	6.99E-243
PGM1	0.66	93.2	1.52E-242
C11orf54	0.65	93.2	1.03E-238
PCBD1	0.87	95	2.12E-238
NME3	0.76	98.2	2.27E-237
BET1	0.62	94.5	8.54E-237
S100A6	1.39	99.5	1.36E-236
GAMT	0.63	90.6	1.72E-233
MT1X	2.15	95.6	2.74E-233
FHL1	0.73	85.2	4.16E-233
HEBP1	0.85	93.6	1.31E-231
MT-ND4	1.59	98.9	2.11E-231
GCSH	0.59	91.5	3.74E-231
MYC	0.64	95.6	1.23E-230
MRPL17	0.62	94.2	1.46E-230
TPI1	1.31	99.1	1.38E-228

PKM	1.15	98.6	1.50E-227
PGGHG	0.73	90.5	7.85E-227
MT1F	0.93	91.2	9.15E-226
TMEM205	0.7	95.5	1.00E-224
ALDH1A1	0.6	90	1.96E-223
TXN	0.91	97.4	5.83E-223
SPINT2	1.06	95.2	1.16E-219
RCN1	0.77	91.7	4.72E-218
MT2A	1.93	98.5	8.52E-218
RPS27L	0.84	98.3	2.27E-217
CBR1	0.72	93.9	7.18E-216
SHMT2	0.69	94.2	1.35E-215
FAM162A	0.7	96.1	8.39E-213
TMEM243	0.73	97.4	1.40E-211
HINT1	1.32	98	2.81E-208
ENO1	1	98.2	9.07E-208
ALDOA	1.18	99.1	9.63E-206
MZT2A	0.7	96.5	8.98E-204
MZT2B	0.7	96.4	5.29E-202
ADI1	0.85	94.1	1.35E-201
LGALS3BP	0.8	95.3	6.15E-201
TIPARP	0.75	96.1	6.63E-200
NME4	0.7	93.8	7.02E-197
PRDX6	0.83	96.8	1.37E-194
HSP90B1	0.89	98	3.18E-194
METTL26	0.64	97.3	3.98E-194
GLRX	0.86	97.1	2.41E-193
SLPI	0.74	88.2	1.57E-191
MT-CO2	1.35	99.4	2.73E-191
ARL1	0.6	92.7	3.61E-191
P4HA1	0.59	94.5	5.91E-190
GSTP1	-1.36	73	1.28E-185
PDCD5	0.61	94.1	4.38E-184
MLEC	0.63	94.7	8.20E-184
CD63	0.87	98.2	5.33E-182
COX7C	0.63	98.8	4.41E-180
PEBP1	0.83	97.1	1.58E-179
DSTN	0.8	94.5	1.08E-177
MT-ND2	1.35	99.1	3.50E-177
VDAC1	0.86	96.7	3.81E-177
CALU	0.6	92.1	1.44E-176
ARPC1A	0.74	92.1	6.00E-174
ATP1A1	0.67	98.2	1.27E-169
ANXA2	0.97	96.4	4.22E-167
MGST3	0.78	95.2	5.07E-162
PSMB5	0.59	92.3	1.86E-158
MT-ND4L	1.31	99.2	9.04E-157
DDT	0.71	94.8	1.34E-153

KDELR2	0.61	95.8	1.29E-152
DDIT4	0.78	99.7	1.54E-151
CD69	-2.09	71.2	3.20E-150
HLA-DPB1	-2.51	79.4	4.20E-148
DUSP23	0.59	95.5	5.28E-146
VIM	0.9	99.4	9.91E-145
TGFBI	0.77	95.3	5.83E-144
LAMTOR5	0.59	94.8	1.72E-140
TMEM176B	0.82	95.5	2.92E-138
HLA-DPA1	-2.52	79.5	1.35E-134
C12orf57	0.61	95.9	3.20E-132
MT1E	1.13	80	3.23E-130
CANX	0.61	96.5	4.16E-129
PDK4	0.96	98.2	3.06E-123
NME2	0.7	96.8	2.98E-122
POLR2L	0.65	96.5	3.41E-121
CD74	-2.01	98	1.18E-120
SERPINA1	1.43	96.7	9.30E-117
HLA-DRB1	-2.64	86.7	3.39E-113
PGAM1	0.71	95.9	6.69E-109
RPLP0	0.83	98.8	8.71E-108
HLA-DRA	-2.82	83.9	1.26E-103
SKP1	0.61	96.8	9.81E-100
TAGLN2	0.63	97.1	5.34E-97
IGKC	-0.68	67.9	1.07E-92
HLA-DRB5	-2.18	83.8	3.33E-88
DUSP2	-1.58	78.2	5.13E-87
TMED2	0.6	93.3	2.09E-85
LAPTM4A	0.62	94.2	7.28E-84
LDHB	0.59	96.2	1.39E-81
CCL4	-2.45	71.1	2.72E-74
NR4A2	-1.24	72.6	4.90E-63
HMGB2	-0.88	88	6.25E-50
GPR34	-0.99	70.6	1.87E-44
C1QA	-2.31	73.5	1.48E-43
CTSW	-1.63	70.5	3.64E-43
LYZ	-2.39	75.2	4.38E-43
FCGR3A	-0.88	70.2	7.96E-43
GPR183	-1.05	67.7	6.85E-41
LTB	-1.42	70.5	1.35E-40
SLC2A3	-0.92	79.8	6.38E-40
ZNF331	-1.21	76.2	7.74E-40
CD7	-1.7	71.2	3.50E-39
CD83	-1.18	71.2	7.05E-39
PLEK	-0.68	71.1	3.15E-36
FGL2	-0.84	70.9	1.61E-35
HSPA6	-1.37	87.6	5.65E-35
BCL2A1	-1.2	70.9	2.65E-34

MS4A6A	-1.64	72.1	3.42E-34
MS4A7	-1.3	72.3	9.71E-34
TREM2	-1.24	71.2	5.11E-33
C1QC	-2.44	73.9	9.18E-33
KLRC1	-0.9	21.2	1.01E-32
C1QB	-2.45	74.7	1.52E-31
NKG7	-2.11	69.7	4.72E-31
APOE	-2.91	79.2	1.78E-29
GZMB	-0.66	67.3	6.86E-28
CCL5	-2.3	73.3	9.99E-28
CTSB	-1.16	92	6.16E-27
EIF4A3	-0.79	85.2	8.16E-27
KLRB1	-1.68	69.2	3.66E-26
APOC1	-2.49	77.3	7.48E-25
LILRB4	-0.97	71.1	1.82E-23
CST7	-1.45	70.2	6.99E-21
KLF2	-0.89	71.1	2.79E-20
ZFAND2A	-0.94	87.6	6.77E-19
IGHG1	-0.72	37.7	2.50E-18
S100A8	-0.86	71.4	3.48E-18
RGCC	-1.22	69.5	7.99E-18
MNDA	-0.77	71.2	1.28E-17
CCL3	-1.75	69.8	5.07E-17
CYBB	-0.83	70.9	6.40E-17
TRDC	-0.63	30.6	2.95E-16
A2M	-0.87	70.6	3.92E-16
OLR1	-1.02	70.2	6.00E-16
STMN1	-0.85	82	2.92E-15
KLRD1	-0.79	67.3	5.16E-15
FCGR2B	-0.73	70.8	9.32E-14
GZMA	-1.46	68.9	1.46E-13
GZMH	-0.63	67	3.40E-13
MS4A4A	-0.85	70.9	8.40E-13
IFITM1	-1.09	90.9	1.03E-12
CKS2	-0.8	82.9	1.40E-12
MAFB	-1.33	76.1	1.42E-12
CD163	-0.78	65.2	1.52E-12
CD247	-0.95	69.1	2.50E-12
CRIP1	-0.69	82.7	3.90E-12
XCL1	-1.09	68.6	7.16E-12
SLC1A3	-0.94	71.7	1.26E-10
SGK1	-0.98	90.3	1.58E-10
SELENOP	-0.97	77	1.81E-10
IL2RB	-0.71	49.1	1.90E-10
VSIG4	-0.72	70.6	4.95E-10
CCL4L2	-1.87	70.3	1.17E-09
CD2	-1.11	70	1.68E-09
CD3D	-1.13	70	5.07E-09

IFNG	-0.8	66.8	5.58E-09
PLD4	-0.82	71.1	1.16E-08
IGSF6	-0.61	70.9	2.39E-08
BCAS2	-0.6	87.4	3.19E-08
IER3	-0.85	87.9	3.22E-08
ISG15	-0.64	82.1	5.26E-08
TNF	-0.93	71.7	1.69E-07
KRT86	-0.62	67.6	4.97E-07
PRF1	-0.89	68.2	3.26E-06
ZFAND5	-0.63	95.3	4.19E-06
CD14	-1.22	75.2	9.97E-06
TM4SF18	0.91	81.9	0
MUC3A	0.76	74.6	0
GPX8	0.63	77.4	3.22E-294
RBPMS	0.61	80.8	2.29E-282
CLTRN	1.04	78	1.63E-273
LOX	0.77	79.1	1.79E-271
CA12	1.1	83.6	2.14E-269
CTGF	0.63	72.9	1.09E-268
RNF128	0.6	75.1	4.80E-244
USH1C	0.63	80.2	1.18E-226
REG1A	0.96	77.4	1.00E-219
GSTA2	1.16	78	1.45E-219
FKBP10	0.85	76.8	2.44E-211
S100A16	1	84.2	4.26E-208
C1S	1.32	87	1.42E-204
SLC16A4	0.66	79.1	1.34E-197
FABP6	0.97	84.7	1.64E-194
ASS1	0.75	77.4	1.61E-190
TFPI2	0.87	78	1.66E-185
TMEM98	0.59	74.6	2.01E-185
CLDN4	0.6	74.6	1.81E-181
SNCG	0.87	78.5	3.08E-176
HSPB8	1.8	92.7	1.63E-174
NRN1	0.74	84.2	2.63E-171
PDZK1IP1	2.34	94.9	2.32E-168
TMEM45A	0.78	84.2	1.81E-163
FABP7	1.96	89.8	6.59E-159
LTF	1.41	78.5	8.52E-152
TM4SF1	0.94	77.4	6.40E-149
CXCL14	2.31	93.2	7.00E-148
CALD1	0.91	80.8	6.22E-146
CYR61	0.7	73.4	2.41E-145
AOC1	0.79	79.7	2.15E-140
UGT2B7	0.74	81.9	5.87E-140
TFPI	1.3	85.9	1.51E-135
RBP5	1.16	84.2	3.31E-135
MGST1	2.3	97.7	9.27E-132

HP	0.63	69.5	7.40E-131
COL1A2	0.78	74.6	2.25E-130
C19orf33	1.81	92.1	2.30E-130
MT3	1.28	88.1	3.78E-129
CAV2	0.96	84.2	4.92E-126
RARRES2	2.44	97.7	2.66E-123
CP	0.72	78.5	3.14E-123
NDUFA4L2	2.91	98.9	4.97E-123
CD24	2.7	97.7	7.24E-122
NNMT	2.53	98.9	2.24E-121
CLU	2.35	96	1.71E-120
S100A1	1.79	89.3	1.50E-119
TSPAN1	0.71	76.8	8.65E-118
BNIP3	2.46	98.3	1.02E-112
CRYAB	2.72	97.2	1.07E-111
UBD	0.85	80.2	1.30E-111
PLOD2	1.27	88.1	2.89E-111
NPTX2	0.76	78	6.76E-109
CAV1	1.59	91	2.61E-106
KRT19	1.74	93.8	2.69E-106
GAPDH	2	100	3.75E-104
KRT8	1.66	94.9	2.02E-103
DEFB1	1.27	82.5	2.31E-103
CRNDE	0.87	84.7	1.23E-102
ADIRF	2.08	90.4	2.16E-102
CA9	0.6	80.2	1.64E-101
IGFBP5	0.8	75.7	6.58E-101
CFB	0.94	80.8	6.74E-100
RARRES1	0.59	76.3	2.20E-98
MIF	1.9	100	4.23E-98
SFRP2	0.88	71.2	3.26E-96
AKR1C3	1.57	88.7	2.95E-95
BCAM	0.66	75.7	1.55E-92
GNG11	0.7	79.7	1.56E-92
FXYD2	2.51	94.4	6.69E-92
ANPEP	0.85	83.1	1.53E-91
LDHA	1.89	98.9	2.59E-91
PCSK1N	0.62	77.4	2.97E-90
WFDC2	1.47	83.1	1.43E-89
SPARC	1.33	80.8	5.19E-88
GPX3	2.53	94.4	5.54E-88
RPLP0	1.3	100	6.74E-88
HINT1	1.76	99.4	3.68E-87
EFNA1	0.63	75.1	5.55E-86
UCHL1	1.33	88.7	8.96E-84
KRT18	1.7	92.7	1.35E-82
BEX3	1.66	92.7	5.06E-82
RNLS	1.29	85.9	1.54E-79

PDLIM1	1.47	88.1	2.49E-79
TPI1	1.54	100	4.19E-78
ANGPTL4	1.41	88.7	2.51E-77
CCDC146	1.16	87	2.75E-77
GSTA1	0.89	79.1	1.68E-76
MMP7	1.09	72.9	7.66E-76
ALDOA	1.3	100	1.15E-75
SAA2	2.3	80.8	4.83E-75
FSTL1	0.66	68.4	1.60E-74
PTGR1	0.79	83.1	1.36E-72
VCAM1	0.77	85.3	2.21E-72
MAOA	0.73	80.8	7.91E-72
PCBD1	1.44	92.7	4.06E-67
IGFBP6	0.7	76.3	7.46E-66
TNFRSF12A	1.35	88.1	7.55E-66
C1R	0.72	80.2	1.09E-65
C12orf75	1.84	93.8	3.62E-65
IFITM3	1.3	98.3	3.18E-64
KRT7	0.67	71.2	3.26E-64
PGRMC1	1.58	92.7	8.37E-64
S100A10	1.53	100	3.91E-63
CYB5A	1.68	94.4	3.47E-62
HSD3B7	0.7	80.2	7.15E-62
IGFBP7	1.74	87.6	7.94E-60
UQCRQ	1.61	98.9	1.35E-58
S100A6	1.11	98.9	3.11E-58
RHEX	0.65	77.4	4.87E-58
IGFBP3	1.92	85.3	6.06E-58
NOL3	0.81	83.1	6.06E-58
AMACR	0.65	81.9	1.65E-57
PFKP	1.32	87	6.92E-56
NQO1	0.59	78	4.17E-55
RPL41	0.78	100	7.23E-55
SPP1	1.25	96	9.68E-55
ANXA4	1.8	93.8	1.16E-53
RACK1	0.82	99.4	3.20E-53
OCIAD2	1.46	93.2	5.46E-53
EGLN3	0.86	84.7	2.63E-52
TMEM176A	1.8	92.7	2.78E-52
VIM	0.95	99.4	3.73E-52
P4HA2	0.6	79.1	2.27E-51
MT-CO2	-0.9	84.2	6.81E-51
CAVIN3	0.7	81.9	9.48E-51
RNASE4	0.61	79.7	2.33E-50
PRDX6	1.3	96.6	7.25E-50
ENO2	0.87	82.5	4.05E-49
MT-ND4L	-0.71	83.6	1.46E-48
NME2	1.07	96.6	1.85E-48

COX7C	0.88	98.3	3.84E-47
CNN3	0.86	85.3	2.46E-45
MXRA7	0.66	78	8.27E-45
GAMT	0.74	84.7	2.83E-44
SEC61G	1.37	98.9	3.44E-44
DEPP1	1.13	85.3	7.58E-44
GSTP1	-1.21	75.1	9.30E-44
S100A13	1.32	89.8	1.81E-43
SAA1	2.66	84.2	2.14E-43
TIMP1	1.21	93.8	4.04E-43
RPS27L	1.08	95.5	4.69E-43
CYSTM1	1.13	91.5	7.92E-43
SPINT2	1.45	92.7	9.27E-43
CISD1	0.99	86.4	4.79E-42
PFN2	1.01	83.6	7.86E-42
NPM1	0.98	97.7	1.59E-41
PRDX1	1.09	98.9	2.15E-40
RPS23	0.61	100	7.03E-40
ADI1	1.32	91.5	2.06E-39
LAPTM4A	1.23	94.9	7.25E-39
HLA-DPB1	-2.15	76.8	7.41E-39
IGHG1	-0.64	55.4	1.69E-38
HLA-DPA1	-2.13	78	2.43E-38
VDAC1	1.43	94.4	4.22E-38
SERPING1	1.1	87	2.72E-37
RIDA	0.6	79.7	3.17E-37
HSPA6	-2.1	78.5	5.97E-37
CD69	-1.47	73.4	2.88E-35
FN1	0.86	84.7	3.27E-35
CRACR2B	0.62	81.4	7.62E-35
TPM1	1.09	86.4	5.01E-34
RAB13	0.91	81.9	5.29E-34
ENO1	1.02	96.6	8.02E-34
IL1R2	0.7	74	8.79E-34
SPAG4	0.68	80.2	1.00E-33
TCEAL9	0.68	83.6	2.08E-33
IGFBP4	1.21	84.2	5.90E-33
SLPI	1.17	77.4	3.27E-32
BET1	0.74	83.1	3.85E-32
P4HB	1.14	94.9	6.23E-32
TGFBI	0.98	88.1	1.50E-31
SLC22A18	0.74	80.8	2.29E-31
MYL6B	0.62	80.8	6.30E-31
LYZ	-1.7	74.6	1.22E-30
HLA-DRA	-2.2	81.4	1.63E-30
TAGLN2	0.73	96	3.64E-30
CD151	1.21	89.3	4.66E-30
DUSP2	-1.49	75.1	5.13E-30

NUPR1	0.68	84.7	7.25E-30
CKB	0.99	83.1	8.64E-30
C1QA	-1.89	74	9.12E-30
CALU	0.92	82.5	2.56E-29
SMPDL3A	0.7	84.2	3.24E-29
IFT22	0.61	78.5	5.56E-29
SERINC2	0.64	81.9	1.17E-28
PKM	0.88	95.5	1.53E-27
SOD2	1.12	94.9	2.32E-27
MT1E	1.81	81.4	1.18E-25
ANXA2	1.09	94.4	2.04E-25
SERPINA1	1.38	93.8	4.45E-25
C1QB	-2.07	75.1	1.45E-24
NDRG1	0.66	83.1	1.62E-24
CD74	-1.57	97.2	2.22E-24
TBCA	1.04	95.5	7.61E-24
MS4A7	-1.14	74	1.15E-23
HLA-DRB1	-1.91	83.1	1.26E-23
DSTN	0.95	89.3	1.26E-23
TMED2	1.13	93.8	2.07E-23
HMGB2	-0.87	78.5	2.56E-23
RCN1	0.87	84.2	4.67E-23
MT2A	1.11	93.8	7.38E-23
PLIN2	0.98	92.1	7.82E-23
GADD45B	-1.13	85.9	1.05E-22
MAFB	-1.34	74	1.68E-22
NME1	0.67	81.9	1.83E-22
PEBP1	1.01	96	2.08E-22
HCFC1R1	0.92	88.1	4.53E-22
PON2	1	86.4	6.75E-22
LDHB	0.95	96	7.49E-22
RRAD	0.9	79.7	1.50E-21
TMEM176B	1	90.4	1.55E-21
POLR2L	0.89	94.4	1.84E-21
LGALS3BP	0.97	84.7	2.39E-21
CCL4	-2.08	75.1	3.55E-21
AP1S1	0.59	84.7	9.62E-21
TMEM9	0.6	80.2	3.92E-20
SELENOM	0.99	85.9	5.16E-20
ZFAS1	0.88	93.8	7.74E-20
HLA-DRB5	-1.55	79.7	1.77E-19
NDUFC1	0.98	89.8	1.89E-19
MS4A6A	-1.55	74	2.14E-19
BAG3	-1.05	79.7	2.44E-19
HMGN3	0.93	93.8	2.61E-19
ARPC1A	0.85	85.9	3.96E-19
GPR34	-0.95	74	4.32E-19
MZT2A	0.76	89.3	5.18E-19

CBR1	0.6	84.7	1.76E-18
ALDH1A1	0.79	82.5	3.14E-18
PGAM1	0.91	93.8	7.83E-18
DDT	0.9	89.3	2.48E-17
ATP1B1	0.85	85.3	3.00E-17
MPC2	0.98	90.4	3.75E-17
PLEK	-0.65	72.3	4.45E-17
SGK1	-1.05	79.7	1.01E-16
FHL1	0.78	78	1.03E-16
NDUFAB1	0.98	88.1	1.19E-16
HMOX1	-0.86	77.4	1.60E-16
PDCD6	0.88	88.7	1.75E-16
PGK1	0.75	93.8	2.03E-16
PERP	0.7	83.6	2.46E-16
SLC2A3	-0.86	76.8	2.96E-16
NR4A2	-1.08	74	8.20E-16
PDCD5	0.79	84.7	1.31E-15
METTL26	1.02	87.6	1.31E-15
TXNDC17	0.82	86.4	2.41E-15
CD63	0.68	96.6	2.72E-15
HEBP1	0.68	83.1	4.09E-15
APOE	-2.47	78	6.08E-15
C1QC	-2.14	74.6	6.41E-15
RHOC	0.77	91.5	1.28E-14
PLVAP	0.75	52	3.41E-14
NHP2	0.91	88.7	6.47E-14
YBX3	0.67	83.1	7.96E-14
CCL5	-2.02	73.4	2.99E-13
KHK	0.84	81.9	4.26E-13
TMEM243	0.75	87.6	4.32E-13
HSP90B1	0.73	91.5	1.51E-12
DNAJB9	0.74	83.6	1.74E-12
RAN	0.92	94.9	2.49E-12
ISG15	-0.68	75.1	3.30E-12
AC245595.1	0.67	82.5	4.20E-12
ERRFI1	0.8	84.2	4.65E-12
GLRX	0.87	91.5	5.20E-12
CTSW	-1.42	74	5.65E-12
CYB5R3	0.81	86.4	5.90E-12
ZNF331	-1.09	74.6	9.11E-12
GPR183	-0.95	68.4	1.10E-11
LAMTOR5	0.71	89.3	1.11E-11
ECHS1	0.6	87	1.25E-11
MGST3	0.92	87	1.61E-11
IER3	-0.77	79.1	1.86E-11
LILRB4	-0.95	74.6	2.48E-11
MNDA	-0.74	73.4	2.89E-11
RHOB	-0.96	86.4	3.51E-11

NUCB2	0.62	79.1	3.56E-11
PAM	0.74	81.9	4.57E-11
MRPS24	0.73	86.4	7.33E-11
NME4	0.72	84.7	8.53E-11
CD7	-1.67	74.6	9.56E-11
FCGR3A	-0.86	71.8	1.39E-10
FGL2	-0.75	74	2.01E-10
CRYZ	0.72	85.3	2.48E-10
RTL8A	0.66	80.2	3.10E-10
KLRB1	-1.47	73.4	4.97E-10
CXCL10	-0.68	67.8	9.35E-10
EIF4A3	-0.65	80.8	9.49E-10
ZFAND5	-0.6	80.8	1.36E-09
AP3S1	0.59	86.4	1.66E-09
CTSB	-0.94	83.1	2.49E-09
MT1X	1.07	85.3	2.49E-09
SLC1A3	-0.96	74	2.85E-09
KDELR2	0.79	88.7	3.78E-09
TUBA1C	0.72	88.7	4.37E-09
PRDX4	0.8	83.1	5.57E-09
HTATIP2	0.62	84.7	6.40E-09
NKG7	-1.58	73.4	7.81E-09
CD163	-0.72	71.2	1.16E-08
CCNG1	0.62	83.6	1.28E-08
TXN	0.68	90.4	1.39E-08
C11orf54	0.75	81.9	1.65E-08
LTB	-1.25	74	2.00E-08
IFITM1	-0.83	79.7	2.74E-08
CST7	-1.16	73.4	3.22E-08
DNAJA4	-0.65	74	3.26E-08
TALDO1	0.74	89.8	4.93E-08
BCL2A1	-0.88	73.4	8.80E-08
DUSP23	0.64	83.1	2.57E-07
ITM2C	-0.68	72.9	3.42E-07
CYBB	-0.8	72.3	6.17E-07
XCL1	-1	72.3	9.33E-07
FAM162A	0.63	85.9	9.91E-07
TMED9	0.59	88.1	1.26E-06
XCL2	-0.98	70.6	1.97E-06
KLRD1	-0.7	72.3	2.95E-06
FCGR1A	-0.61	74	4.49E-06
CD247	-0.93	72.9	5.33E-06

Abbreviations: Percentage: percentage of fibroblast that express this specific gene.

Table S3 Thirteen differentially expressed fibroblast immune-related genes.

Gene	HR (95%Cl)	P-value
CLDN4	0.254(0.133-0.483)	0
TNFRSF12A	3.252(1.178-8.981)	0.023
DEFB1	0.594(0.377-0.938)	0.025
LTF	0.712(0.531-0.955)	0.023
SAA2	1.384(1.120-1.710)	0.003
SAA1	1.473(1.218-1.780)	0
MDK	2.976(1.572-5.630)	0.001
SLPI	1.328(1.051-1.678)	0.018
CANX	0.118(0.028-0.496)	0.004
HLA-DRA	0.280(0.086-0.909)	0.034
LYZ	0.626(0.418-0.935)	0.022
A2M	0.276(0.113-0.675)	0.005
ISG15	3.843(1.261-11.718)	0.018

Abbreviation: HR: Hazard Ratio; CL: Confidence level.

Table S4 Six differentially expressed FIGs.

Gene	Coefficient
CLDN4	-0.666898209
LTF	-0.318111131
SAA1	0.260840889
MDK	0.741319658
HLA-DRA	-1.30194155
ISG15	0.793055552

Abbreviation: FIGs: fibroblast immune related genes.

Table S5 The most enriched 5 GSEA pathways for different risk groups.

Pathways	Group
**h.all.v2023.2.Hs.symbols.gmt**	
HALLMARK_PROTEIN_SECRETION	low
HALLMARK_FATTY_ACID_METABOLISM	low
HALLMARK_ANDROGEN_RESPONSE	low
HALLMARK_HEME_METABOLISM	low
HALLMARK_OXIDATIVE_PHOSPHORYLATION	low
HALLMARK_IL6_JAK_STAT3_SIGNALING	high
HALLMARK_ALLOGRAFT_REJECTION	high
HALLMARK_ANGIOGENESIS	high
HALLMARK_EPITHELIAL_MESENCHYMAL_TRANSITION	high
HALLMARK_COAGULATION	high
**c2.cp.kegg.Hs.symbols.gmt**	
KEGG_ADIPOCYTOKINE_SIGNALING_PATHWAY	low
KEGG_ARACHIDONIC_ACID_METABOLISM	low
KEGG_ASCORBATE_AND_ALDARATE_METABOLISM	low
KEGG_BASAL_CELL_CARCINOMA	low
KEGG_CITRATE_CYCLE_TCA_CYCLE	low
KEGG_STEROID_HORMONE_BIOSYNTHESIS	high
KEGG_TYPE_II_DIABETES_MELLITUS	high
KEGG_TYROSINE_METABOLISM	high
KEGG_VALINE_LEUCINE_AND_ISOLEUCINE_DEGRADATION	high
KEGG_VIBRIO_CHOLERAE_INFECTION	high
**c2.cp.wikipathways.v2023.2.Hs.symbols.gmt**	
WP_PROXIMAL_TUBULE_TRANSPORT	low
WP_LEUCINE_ISOLEUCINE_AND_VALINE_METABOLISM	low
WP_TYPE_II_DIABETES_MELLITUS	low
WP_ERK_PATHWAY_IN_HUNTINGTON_39_S_DISEASE	low
WP_NOTCH1_REGULATION_OF_ENDOTHELIAL_CELL_CALCIFICATION	low
WP_GLUCURONIDATION	high
WP_TAMOXIFEN_METABOLISM	high
WP_STATIN_INHIBITION_OF_CHOLESTEROL_PRODUCTION	high
WP_FAMILIAL_HYPERLIPIDEMIA_TYPE_4	high
WP_FAMILIAL_HYPERLIPIDEMIA_TYPE_1	high
**c5.go.Hs.symbols.gmt**	
GOBP_SPLICEOSOMAL_TRI_SNRNP_COMPLEX_ASSEMBLY	low
GOBP_REGULATION_OF_SYSTEMIC_ARTERIAL_BLOOD_PRESSURE_BY_CIRCULATORY_RENIN_ANGIOTENSIN	low
GOCC_U5_SNRNP	low
GOBP_SPLICEOSOMAL_SNRNP_ASSEMBLY	low
GOCC_SPLICEOSOMAL_TRI_SNRNP_COMPLEX	low
GOCC_IMMUNOGLOBULIN_COMPLEX	high

GOBP_REGULATION_OF_LIPOPROTEIN_LIPASE_ACTIVITY	high
GOMF_IMMUNOGLOBULIN_RECEPTOR_BINDING	high
GOCC_IMMUNOGLOBULIN_COMPLEX_CIRCULATING	high
GOCC_HIGH_DENSITY_LIPOPROTEIN_PARTICLE	high
**c7.immunesigdb.v2023.2.Hs.symbols.gmt**	
GSE40274_FOXP3_VS_FOXP3_AND_HELIOS_TRANSDUCED_ACTIVATED_CD4_TCELL_DN	low
GSE40493_BCL6_KO_VS_WT_TREG_UP	low
GSE1791_CTRL_VS_NEUROMEDINU_IN_T_CELL_LINE_6H_DN	low
GSE17974_CTRL_VS_ACT_IL4_AND_ANTI_IL12_24H_CD4_TCELL_UP	low
GSE40666_UNTREATED_VS_IFNA_STIM_STAT4_KO_EFFECTOR_CD8_TCELL_90MIN_DN	low
GSE39110_DAY3_VS_DAY6_POST_IMMUNIZATION_CD8_TCELL_DN	high
GSE14415_NATURAL_TREG_VS_TCONV_DN	high
GSE15750_DAY6_VS_DAY10_TRAF6KO_EFF_CD8_TCELL_UP	high
GSE15750_DAY6_VS_DAY10_EFF_CD8_TCELL_UP	high
GSE45365_HEALTHY_VS_MCMV_INFECTION_CD11B_DC_DN	high
**c7.vax.v2023.2.Hs.symbols.gmt**	
KENNEDY_PBMC_DRYVAX_AGE_18_50YO_STIMULATED_VS_UNSTIMULATED_1_TO_48MO_TOP_DEG_UP	low
HOFT_CD4_POSITIVE_ALPHA_BETA_MEMORY_T_CELL_BCG_VACCINE_AGE_18_45YO_56D_TOP_100_DEG_AFTER_IN_VITRO_RE_STIMULATION_UP	low
HOEK_NEUTROPHIL_2011_2012_TIV_ADULT_1DY_UP	low
ERWIN_COHEN_BLOOD_VACCINE_TC_83_AGE_23_48YO_VACCINATED_VS_CONTROL_2DY_DN	low
THAKAR_PBMC_INACTIVATED_INFLUENZA_AGE_21_30YO_NONRESPONDER_28DY_UP	low
LI_PBMC_MENOMUNE_A_C_Y_W_135_AGE_18_45YO_3DY_UP	high
SOBOLEV_PBMC_PANDEMRIX_AGE_18_64YO_RESPONDERS_VS_NONRESPONDERS_7DY_UP	high
HOEK_PBMC_INACTIVATED_INFLUENZA_ADULT_7DY_UP	high
LI_PBMC_MENACTRA_AGE_18_45YO_ANTI_DT_ANTIBODY_CORRELATION_PROFILE_3DY_DN	high
HOWARD_NK_CELL_INACT_MONOV_INFLUENZA_A_INDONESIA_05_2005_H5N1_AGE_18_49YO_3DY_UP	high

Abbreviations: GSEA: Gene Set Enrichment Analysis.

Table S6 Antineoplastic drug sensitivity information (no obviously sensitive group).

Drug	High risk group	Low risk group	P-value
	ISS (25%-75%)	ISS (25%-75%)	
Camptothecin	0.10(0.06-0.20)	0.12(0.07-0.19)	0.170
Cisplatin	4.46(3.54-5.54)	4.83(3.99-5.60)	0.011
Cytarabine	2.64(2.01-3.48)	2.60(2.01-3.30)	0.542
Docetaxel	0.01(0.01-0.02)	0.01(0.01-0.02)	0.306
Navitoclax	3.16(1.73-4.18)	2.96(1.87-3.98)	0.413
Vorinostat	2.43(1.91-2.89)	2.40(2.06-2.76)	0.932
Nilotinib	5.22(4.63-5.95)	5.07(4.66-5.55)	0.057
Olaparib	6.35(5.58-7.02)	6.08(5.54-6.65)	0.024
KU-55933	6.46(6.35-6.56)	6.48(6.40-6.56)	0.044
Staurosporine	0.06(0.03-0.10)	0.07(0.04-0.11)	0.010
PLX-4720	6.33(5.96-6.82)	6.44(5.99-6.80)	0.554
NU7441	3.83(3.56-4.09)	3.77(3.53-4.01)	0.061
Doramapimod	6.55(6.38-6.75)	6.48(6.33-6.64)	0.001
Mirin	6.67(6.15-7.32)	6.86(6.45-7.21)	0.022
ZM447439	4.34(4.06-4.61)	4.31(4.02-4.51)	0.142
Alisertib	2.88(2.21-3.72)	2.72(2.26-3.32)	0.177
Palbociclib	5.16(4.52-5.78)	5.34(4.89-5.86)	0.006
Dactolisib	0.23(0.15-0.39)	0.25(0.18-0.37)	0.018
PD0325901	1.31(1.04-1.72)	1.38(1.15-1.74)	0.117
Obatoclax Mesylate	2.26(1.96-2.65)	2.26(2.01-2.59)	0.865
5-Fluorouracil	6.55(5.51-7.59)	6.90(6.17-7.83)	0.001
Paclitaxel	0.07(0.04-0.16)	0.08(0.04-0.14)	0.794
Crizotinib	4.70(4.27-5.16)	4.57(4.24-5.00)	0.622
Rapamycin	0.15(0.09-0.25)	0.15(0.10-0.22)	0.485
Sorafenib	3.96(3.43-4.54)	3.79(3.36-4.23)	0.011
Irinotecan	3.60(2.89-4.42)	3.82(3.21-4.63)	0.014
Oxaliplatin	5.53(4.81-6.17)	5.31(4.71-5.81)	0.014
PRIMA-1MET	6.59(5.65-7.38)	6.48(5.92-7.15)	0.915
Niraparib	6.19(5.50-6.81)	6.12(5.62-6.69)	0.993
MK-1775	1.48(1.16-1.90)	1.33(1.13-1.68)	0.010
Gemcitabine	0.53(0.26-0.93)	0.56(0.35-1.07)	0.038
Bortezomib	0.01(0.01-0.01)	0.01(0.01-0.01)	0.604
GSK269962A	4.26(3.99-4.47)	4.30(4.11-4.47)	0.096
Tamoxifen	5.15(4.85-5.46)	5.16(4.93-5.41)	0.792
Fulvestrant	4.22(3.97-4.58)	4.24(4.01-4.54)	0.633
EPZ004777	7.34(6.92-7.96)	7.30(6.96-7.81)	0.580
YK-4-279	3.33(2.49-4.13)	3.35(2.65-4.00)	0.568
Talazoparib	4.47(3.51-5.44)	4.62(3.83-5.40)	0.194
Trametinib	1.50(0.92-1.89)	1.53(1.13-2.07)	0.034
Dabrafenib	6.56(6.00-7.01)	6.70(6.22-7.13)	0.009
Temozolomide	8.60(8.15-9.11)	8.51(8.10-8.96)	0.113
AZD5438	3.20(2.71-4.11)	3.05(2.60-3.51)	0.003
AZD1208	7.69(7.14-8.24)	7.57(7.15-7.93)	0.013
AZD1332	5.58(5.09-5.99)	5.56(5.21-5.92)	0.150

Linsitinib	5.51(3.01-6.03)	5.38(5.01-5.79)	0.046
Epirubicin	0.43(0.23-0.71)	0.42(0.29-0.61)	0.969
Cyclophosphamide	7.46(7.15-7.84)	7.33(7.05-7.62)	0.001
Pevonedistat	1.59(1.05-2.41)	1.43(0.98-2.02)	0.065
Sapitinib	5.78(5.41-6.15)	5.70(5.38-5.98)	0.028
Uprosertib	4.33(3.80-4.80)	4.34(3.98-4.77)	0.318
Lapatinib	4.31(3.91-4.69)	4.48(4.13-4.77)	0.004
Luminespib	0.12(0.06-0.21)	0.14(0.09-0.23)	0.001
EPZ5676	7.99(7.67-8.44)	7.84(7.57-8.17)	0.001
SCH772984	3.85(3.18-4.48)	3.97(3.51-4.45)	0.103
Leflunomide	7.18(6.91-7.53)	7.13(6.88-7.38)	0.104
Entinostat	3.19(2.49-3.94)	3.46(2.90-3.94)	0.006
VE-822	4.84(4.33-5.32)	4.79(4.38-5.22)	0.922
CZC24832	7.23(6.98-7.53)	7.31(7.10-7.54)	0.020
AZD5582	3.29(2.54-4.21)	3.07(2.46-3.77)	0.076
I-BET-762	4.98(4.30-5.47)	4.70(4.23-5.17)	0.003
OTX015	3.73(3.03-4.33)	3.64(3.11-4.10)	0.426
GSK343	4.11(3.81-4.45)	4.08(3.85-4.35)	0.671
Entospletinib	5.42(4.98-5.80)	5.29(4.99-5.67)	0.187
Ribociclib	5.58(5.11-5.70)	5.58(5.49-5.67)	0.732
Picolinici-acid	7.37(7.08-7.79)	7.29(7.07-7.56)	0.009
AZD5153	2.73(2.20-3.24)	2.66(2.24-3.02)	0.181
CDK9_5038	0.12(0.07-0.27)	0.10(0.06-0.17)	0.010
Eg5_9814	0.06(0.03-0.13)	0.05(0.03-0.08)	0.002
ERK_2440	3.94(3.17-4.84)	3.72(3.13-4.25)	0.018
ERK_6604	4.96(4.92-4.99)	4.96(4.93-4.99)	0.540
IRAK4_4710	7.11(6.75-7.55)	7.02(6.70-7.31)	0.028
JAK1_8709	5.97(5.60-6.53)	5.94(5.59-6.27)	0.111
PAK_5339	3.58(3.36-3.83)	3.4(3.30-3.73)	0.011
VSP34_8731	3.55(3.02-4.22)	3.33(2.88-3.86)	0.002
Selumetinib	5.934929633	6.10(5.52-6.73)	0.079
IGF1R_3801	2.53(1.94-3.16)	2.62(2.20-3.09)	0.090
JAK_8517	4.26(3.51-5.10)	4.38(3.80-4.95)	0.199
Oxaliplatin	5.53(4.81-6.17)	5.31(4.73-5.81)	0.014
Topotecan	1.03(0.61-1.49)	1.09(0.76-1.48)	0.060
Teniposide	1.40(0.81-2.29)	1.25(0.82-1.79)	0.114
Mitoxantrone	1.45(1.00-2.16)	1.38(1.03-1.82)	0.070
Dactinomycin	0.12(0.07-0.19)	0.10(0.07-0.14)	0.016
Fludarabine	7.14(6.37-8.04)	7.25(6.49-7.84)	0.882
Fulvestrant	4.22(3.97-4.58)	4.24(4.01-4.54)	0.631
Vincristine	0.21(0.10-0.50)	0.17(0.10-0.28)	0.014
Docetaxel	0.01(0.01-0.02)	0.01(0.01-0.02)	0.306
Podophyllotoxin bromide	0.54(0.40-0.84)	0.54(0.42-0.73)	0.969
Elephantin	5.04(4.15-5.87)	4.71(4.23-5.23)	0.004
Sabutoclax	0.70(0.53-0.97)	0.72(0.53-0.93)	0.968
BDP-00009066	3.50(3.11-3.83)	3.51(3.21-3.82)	0.405
Venetoclax	3.18(2.78-3.68)	3.26(2.88-3.59)	0.721

ABT737	3.28(1.82-4.31)	3.57(2.52-4.37)	0.102
Dactinomycin	0.12(0.07-0.19)	0.10(0.07-0.14)	0.016
AGI-5198	6.64(6.36-7.00)	6.79(6.56-7.00)	0.005
AZD6738	2.99(2.52-3.56)	3.09(2.76-3.50)	0.06
Ipatasertib	5.04(4.59-5.45)	5.21(4.76-5.60)	0.008
GDC0810	7.09(6.83-7.41)	7.05(6.84-7.35)	0.182
Telomerase Inhibitor IX	1.35(1.04-1.75)	1.33(1.06-1.69)	0.882
MIRA-1	7.87(7.42-8.25)	7.78(7.33-8.14)	0.174
NVP-ADW742	4.08(3.74-4.50)	3.92(3.53-4.30)	0.001
Savolitinib	3.84(3.61-4.14)	3.86(3.57-4.12)	0.771
MIM1	5.69(5.13-6.16)	5.58(5.22-5.98)	0.220
WEHI-539	5.15(4.53-5.76)	5.07(4.46-5.51)	0.060
BPD-00008900	6.52(6.01-691)	6.49(6.17-6.85)	0.589
Foretinib	1.83(1.55-2.18)	1.79(1.60-2.06)	0.669
Pyridostatin	4.84(4.36-5.24)	4.86(4.45-5.28)	0.261
AMG-319	6.96(6.60-7.30)	6.92(6.68-7.17)	0.589
VX-11e	4.14(3.57-4.62)	4.29(3.78-4.73)	0.007
Uprosertib	4.33(3.90-4.80)	4.349342875	0.318
LJI308	7.24(6.85-7.65)	7.36(7.06-7.71)	0.019
AZ6102	3.67(3.29-4.00)	3.49(3.25-3.82)	0.003
GSK591	6.60(6.16-7.06)	6.50(6.12-7.02)	0.148
VE821	5.90(5.38-6.41)	5.86(5.48-6.30)	0.895
AZD6482	4.50(4.20-4.93)	4.59(4.23-4.89)	0.454
AT13148	5.14(4.76-5.79)	5.10(4.67-5.74)	0.496
BMS-754807	1.27(0.91-1.58)	1.23(0.98-1.46)	0.517
JQ1	3.54(3.06-4.01)	3.38(2.94-3.92)	0.088

Abbreviation: ISS: Imputed sensitivity score.

Table S7 Antineoplastic drug sensitivity information (sensitive group: low).

Drug	High risk group	Low risk group	P-value
	ISS (25%-75%)	ISS (25%-75%)	
Gefitinib	4.80(4.46-5.14)	4.62(4.31-4.93)	0
Axitinib	4.54(4.30-4.87)	4.42(4.15-4.63)	0
SB216763	7.49(7.17-7.96)	7.35(7.10-7.65)	0
Afatinib	2.87(2.58-3.15)	2.74(2.51-2.97)	0
Wee1 Inhibitor	3.01(2.62-3.75)	2.81(2.47-3.35)	0
PD173074	5.99(5.43-6.50)	5.65(5.21-6.11)	0
RO-3306	4.16(3.83-4.45)	3.92(3.62-4.25)	0
BI-2536	1.25(0.95-1.60)	1.09(0.83-1.42)	0
GSK1904529 A	6.42(5.94-6.91)	5.93(5.54-6.37)	0
Tozasertib	4.33(3.90-4.75)	4.20(3.77-4.46)	0
Erlotinib	3.96(3.60-4.30)	3.73(3.47-4.04)	0
Dinaciclib	0.08(0.06-0.15)	0.06(0.04-0.09)	0
SB505124	3.40(3.25-3.62)	3.30(3.18-3.44)	0
Daporinad	0.02(0.01-0.04)	0.01(0.01-0.02)	0
BMS-345541	5.06(4.50-5.76)	4.42(4.01-5.04)	0
IAP_5620	7.48(7.01-8.08)	7.03(6.64-7.57)	0
Ruxolitinib	7.10(6.77-7.49)	6.81(6.52-7.13)	0
LCL161	7.29(6.95-7.63)	6.85(6.52-7.28)	0
IWP-2	4.15(3.87-4.41)	3.98(3.72-4.19)	0
OSI-027	6.77(6.67-6.88)	6.68(6.59-6.78)	0
LGK974	5.94(5.52-6.41)	5.61(5.27-6.01)	0
GSK2606414	5.50(5.08-5.95)	5.27(4.98-5.61)	0
PFI3	7.59(7.33-7.90)	7.46(7.26-7.68)	0
PCI-34051	6.49(6.02-7.18)	6.25(5.88-6.64)	0
Wnt-C59	6.20(5.91-6.63)	5.88(5.66-6.15)	0
RVX-208	6.91(6.62-7.24)	6.78(6.54-7.02)	0
ML323	6.61(6.29-6.96)	6.24(5.99-6.54)	0
AGI-6780	6.01(5.65-6.48)	5.76(5.52-6.05)	0
CDK9_5576	0.72(0.51-1.20)	0.58(0.45-0.81)	0
AZD5991	6.39(5.45-7.27)	5.86(5.07-6.60)	0
TAF1_5496	5.79(5.28-6.33)	5.34(4.86-5.80)	0
AZD4547	4.29(3.94-4.77)	4.12(3.77-4.41)	0
Ibrutinib	6.78(6.31-7.31)	6.17(5.67-6.63)	0
Zoledronate	5.53(5.12-5.97)	5.27(5.00-5.63)	0
Acetalax	7.19(6.58-7.90)	6.90(6.34-7.34)	0
Carmustine	8.95(8.59-9.37)	8.56(8.25-8.96)	0
Nelarabine	8.76(8.15-9.44)	8.42(7.88-8.98)	0
Dihydrorotenone	1.98(1.68-2.31)	1.57(1.31-7.84)	0
Gallibiscoquin	3.93(3.60-4.39)	3.61(3.36-3.94)	0

azole			
Sinularin	5.32(4.82-5.99)	4.84(4.41-5.32)	0
LY2109761	7.64(7.08-8.25)	7.03(6.61-7.50)	0
OF-1	6.16(5.76-6.78)	5.59(5.17-6.00)	0
MN-64	6.86(6.49-7.40)	6.66(6.26-6.98)	0
KRAS (G12C) Inhibitor-12	6.41(5.80-7.24)	5.91(5.44-6.52)	0
Ulixertinib	4.19(3.83-4.69)	3.99(3.59-4.32)	0
AZD3759	4.00(3.72-4.33)	3.84(3.60-4.11)	0
Osimertinib	2.91(2.52-3.22)	2.59(2.27-2.89)	0
Cediranib	3.32(2.97-3.75)	3.10(2.82-3.36)	0
I-BRD9	6.40(6.03-6.80)	6.22(5.77-6.53)	0
P22077	6.77(6.19-7.34)	5.99(5.56-6.57)	0
UMI-77	4.06(3.59-4.81)	3.76(3.30-4.19)	0
BIBR-1532	7.20(6.75-7.79)	6.91(6.53-7.23)	0

Abbreviation: ISS： Imputed sensitivity score.

Table S8 Antineoplastic drug sensitivity information (sensitive group: high).

Drug	High risk group	Low risk group	P-value
	ISS (25%-75%)	ISS (25%-75%)	
Vinblastine	0.02(0.01-0.05)	0.03(0.02-0.06)	0
AZD7762	0.89(0.65-1.24)	1.16(0.90-1.46)	0
Nutlin-3a (-)	6.45(5.61-7.38)	6.83(6.32-7.44)	0
MK-2206	4.26(3.87-4.73)	4.43(4.13-4.77)	0
Pictilisib	2.11(1.75-2.60)	2.47(2.14-2.89)	0
AZD8055	0.84(0.79-0.89)	0.88(0.84-0.92)	0
Dasatinib	2.50(1.64-3.29)	2.84(2.22-3.48)	0
BMS-536924	3.10(2.71-3.50)	3.31(2.97-3.61)	0
PF-4708671	5.60(5.35-5.78)	5.70(5.50-5.87)	0
AZ960	2.90(2.37-3.45)	3.21(2.85-3.71)	0
XAV939	6.17(5.91-6.49)	6.48(6.26-6.73)	0
AZD2014	3.01(2.61-3.44)	3.24(2.97-3.59)	0
Alpelisib	4.97(4.48-5.40)	5.32(4.90-5.75)	0
Taselisib	3.00(2.29-3.65)	3.43(2.77-3.86)	0
WZ4003	5.23(4.85-5.64)	5.49(5.12-5.81)	0
PRT062607	4.57(4.26-4.94)	4.81(4.55-5.13)	0
ULK1_4989	3.14(2.61-3.77)	3.66(3.10-4.21)	0
MG-132	0.25(0.21-0.29)	0.26(0.23-0.31)	0
Buparlisib	1.75(1.53-2.02)	1.86(1.70-2.09)	0
Afuresertib	3.63(3.04-4.16)	3.95(3.43-4.28)	0
AZD5363	4.19(3.73-4.55)	4.43(4.08-4.80)	0
AZD8186	4.62(4.35-4.99)	4.82(4.54-5.13)	0
GNE-317	1.32(1.15-1.54)	1.52(1.37-1.69)	0
GSK2578215A	6.97(6.70-7.30)	7.11(6.95-7.32)	0
WIKI4	5.30(5.14-5.51)	5.40(5.26-5.57)	0
Sepantronium bromide	0.02(0.01-0.04)	0.01(0.01-0.02)	0
MK-8776	4.51(3.99-5.01)	4.74(4.38-5.05)	0
Vinorelbine	0.05(0.02-0.12)	0.07(0.03-0.14)	0
Vinblastine	0.03(0.01-0.05)	0.04(0.02-0.07)	0

Abbreviation: ISS： Imputed sensitivity score.
